# Decoding the Oncogenic Role of USP22 Through Pan‐Cancer Genomic and Epigenetic Analysis

**DOI:** 10.1002/cnr2.70572

**Published:** 2026-05-13

**Authors:** Uma Devi A., Prakash Kumar Shukla

**Affiliations:** ^1^ Center for Bioseparation Technology VIT Vellore India

## Abstract

**Background:**

Ubiquitin‐specific protease 22, an important catalytic component of the human SAGA (Spt‐Ada‐GcN5 Acetyltransferase) complex, regulates the deubiquitination and methylation of histones, which in turn influences gene expression. Its overexpression alters gene regulation, transcription, cancer progression, and therapy resistance. Its role is increasingly being noticed in cancers.

**Aims:**

To conducted a pan‐cancer analysis across multiple malignancies, as it allows for a comprehensive assessment of USP22 expression, regulation, and clinical impact.

**Methods:**

The Human Protein Atlas, UALCAN, and Timer 2.0 were used to examine USP22 expression at the gene and protein levels in 33 TCGA cancer types. Furthermore, various tools have been employed to study genetic changes, overall survival (OS), disease‐free survival (DFS), DNA methylation profiles, and immune associations. Gene correlation and protein–protein interaction were examined.

**Results:**

USP22 expression level was observed to be significantly higher in 13 distinct cancer types among the 33 TCGA cancer types. Along with the pathological stages of the TCGA sample, it showed overexpression in the histological subtypes, TP53 mutant stages, and tumor grade in numerous cancers compared to the control. According to the study, elevated USP22 expression was linked to a worse chance of survival and a lower OS rate in several types of cancer. High expression of USP22 is linked to a poor immunosuppressive microenvironment, and the CpG‐aggregated methylation analysis shows that the gene is significantly hypomethylated in tumor samples, which is highly associated with its known upregulation in cancer.

**Conclusion:**

USP22 may have potential relevance in cancer, and the pathways associated with it could offer possible targets for therapeutic intervention.

## 1 Introduction

The posttranslational modification influences the functions and destination of most proteins. Ubiquitination is one of the modifications that involves the addition of a small protein ubiquitin, which consists of 76 amino acids, to the substrate lysine [1]. Three enzymes function sequentially to ubiquitinate target substrates: (1) a ubiquitin (Ub)‐activating enzyme (E1) charges ubiquitin, (2) an Ub‐conjugating enzyme (E2) transfers ubiquitin to substrate lysine with the help of (3) an Ub ligase (E3) [2]. The substrate/target protein ubiquitination status is maintained in equilibrium by deubiquitinases (DUBs), which hydrolyze ubiquitinated substrate at isopeptide bonds [3]. The human genome encodes about 100 DUBs [4] and is divided into eight subfamilies according to the structure of their catalytic domain. The most widely distributed DUB subfamily is made up of USPs, which include 58 vertebrate members [5]. DUBs control cellular differentiation, cell cycle regulation, endocytosis, membrane receptor signaling, DNA‐dependent activities, protein quality control, and cell survival and death, regulating numerous physiological functions [5, 6].

The chromatin that houses the cellular genome needs to be remodeled during transcription, DNA replication, and cell division. Several posttranslational modifications that aid in chromatin remodeling target the protruding tails of these nucleosomes. Recently, several histone DUBs with varying specificities for H2A or H2B have been identified, one of which is USP22 [7]. USP22 is a member of the biggest subfamily of deubiquitinases, known as ubiquitin‐specific processing proteases (USPs), whose members are also referred to as ubiquitin‐binding proteins (UBPs) in yeast [8]. In contrast to other deubiquitinating enzymes, USP22/UBP8 zinc‐finger ubiquitin‐binding domain (ZnF‐UBP) does not directly bind to ubiquitin and lacks ubiquitin tail‐binding pockets. Instead, it binds to Ataxin 7‐like protein 3 (ATXN7L3)/sgf11, and ENY2 transcription and export complex 2 subunit/sucrose synthase 1 (ENY2/Sus1), and Ataxin 7/Spt‐Ada‐Gcn5‐acetyltransferase (SAGA)‐associated factor 73 (ATXN7/Sgf73) to form the firmly bound deubiquitinase module (DUBm) of SAGA [9, 10]. Histones and nonhistone proteins have been identified as the substrate of USP22/Ubp8. USP22, the primary component of hSAGA DUBm, is essential for histone control by deubiquitinating H2BK120. The ubiquitination level of H2B affects the levels of histone 3 (H3) methylation at specific sites, which plays an important role in active transcription [11, 12]. It also stabilizes the nonhistone substrates like B cell‐specific Moloney murine leukemia virus integration site 1 (BMI‐1), c‐Myc, cyclin D1, NFATc2, far‐upstream element–binding protein 1 (FBP1), and sirtuin 1 (SIRT1) [9]. There is solid evidence indicating that USP22 plays a role in transcriptional regulation, cell‐cycle progression, and carcinogenesis; the exact mechanisms through which USP22 affects these processes remain unclear [9].

USP22 was shown to be upregulated and associated with a poor prognosis in 14 solid tumors, including breast cancer, bladder cancer, cervical cancer, colorectal cancer, esophageal carcinoma (ESCA), gliomas, gastric cancer, lung cancer, liver cancer, oral squamous cell carcinoma, salivary duct carcinoma, salivary adenoid cystic carcinoma, pancreatic cancer, prostate cancer, and thyroid cancer [9]. In cancer cells, USP22 is thought to be a crucial regulator of the cell cycle and telomere maintenance, permitting the unchecked growth of tumor cells with numerous DNA replication mistakes. Currently, there is no specific inhibitor available for USP22 in the market, which suggests that cell cycle inhibitors could potentially be more effective in addressing malignancies associated with USP22 overexpression [9].

Given USP22 rising relevance as a possible therapeutic target in oncology, we examined its expression levels across 33 cancer types using data from The Cancer Genome Atlas (TCGA). USP22 has been associated with multiple processes, positioning it as a key target for drug discovery initiatives.

## 2 Materials and Methods

Several bioinformatics tools have been utilized for the current investigation, including Tumor Immune Estimation Resource 2.0 (TIMER 2.0) (http://timer.cistrome.org/), University of Alabama Cancer Database (UALCAN) website (https://ualcan.path.uab.edu/analysis.html), Human Protein Atlas (HPA) database (https://www.proteinatlas.org/), Gene Expression Profiling Interactive analysis 2 (GEPIA2) database (http://gepia2.cancer‐pku.cn), and cBioPortal database (https://www.cbioportal.org/).

To analyze the gene expression of USP22 across cancer types, we utilized Timer 2.0, which integrates expression data from TCGA and the Genome Tissue Expression (GTEx) database to provide expression between tumor and adjacent normal tissue. A total of 33 cancer types: Adrenocortical carcinoma (ACC), breast invasive carcinoma (BRCA), bladder urothelial carcinoma (BLCA), colon adenocarcinoma (COAD), lymphoid neoplasm diffuse large B‐cell lymphoma (DLBC), ESCA, glioblastoma multiforme (GBM), brain lower grade glioma (LGG), head and neck squamous cell carcinoma (HNSC), kidney renal clear cell carcinoma (KIRC), kidney renal papillary cell carcinoma (KIRP), kidney chromophobe (KICH), acute myeloid leukemia (LAML), lung squamous cell carcinoma (LUSC), liver hepatocellular carcinoma (LIHC), lung adenocarcinoma (LUAD), ovarian serous cystadenocarcinoma (OV), prostate adenocarcinoma (PRAD), pancreatic adenocarcinoma (PAAD), rectum adenocarcinoma (READ), stomach adenocarcinoma (STAD), skin cutaneous melanoma (SKCM), thyroid carcinoma (THCA), thymoma (THYM), testicular germ cell tumor (TGCT), uterine corpus endometrial carcinoma (UCEC), and uterine carcinosarcoma (UCS), were chosen from the TCGA dataset (v42.0, January 30, 2025) based on the availability of both expression and clinical survival data. TIMER 2.0 normalizes the gene expression levels through log_2_ (TPM + 1) transformation, and using the Wilcoxon rank‐sum test, statistical significance was evaluated, and a *p* value of < 0.05 was deemed statistically significant. To analyze the pan‐cancer protein expression of USP22, the UALCAN web server's Clinical Proteomic Tumor Analysis Consortium (CPTAC) module was employed. Additionally, the Cancer Atlas module of HPA provides protein expression data for USP22 across various tumors, utilizing immunohistochemistry (IHC) staining with the antibody HPA044980 (Atlas Antibodies, RRID: AB_10794503).

We used the UALCAN web server [13, 14], which links the data to TCGA and CPTAC, to investigate USP22 expression across different cancer clinical stages, tumor grades, and histological subtypes. A box plot created with UALCAN was used to display the difference in expression, and the server uses one‐way ANOVA to determine the significance (*p* < 0.05 was deemed significant).

Survival analysis was done by utilizing default settings from the GEPIA2 online tool using data from GTEx and TCGA. Kaplan–Meier (KM) plots were used to evaluate disease‐free survival (DFS) and overall survival (OS) in groups with low and high expression. GEPIA2 evaluates significance using a log‐rank test and hazard ratio (HR) with a 95% confidence interval (CI) estimated using a univariate Cox proportional model. A *p* value < 0.05 indicated statistical significance. Using R software version 4.5.3, multivariate Cox proportional hazards regression analysis was carried out to further evaluate if USP22 is an independent prognostic factor. The gene expression, related survival, and clinical data were obtained from publicly available datasets using the UCSC Xena browser (https://xenabrowser.net/datapages/). The gene expression data were obtained as RNA‐Seq data processed using the RSEM normalization method and expressed in transcript per million (TPM). Age, gender (male vs. female), tumor stage (late vs. early), and tumor grade (high vs. low) were all included in the multivariate Cox regression analysis. Sample IDs were used to merge the clinical and gene expression datasets, and duplicate samples were removed prior to analysis. The reference groups were low‐grade and early‐stage, and categorical data were transformed into factors. Then, HRs with 95% CIs were calculated. KM plots were developed, and forest plots were used to illustrate multivariate Cox regression analysis. A *p* value of less than 0.05 is deemed statistically significant.

The USP22 genetic alteration study was carried out utilizing the cBioPortal database, which combines genomic information from larger‐scale cancer studies and TCGA. The prevalence and kinds of genetic changes, such as mutations, amplifications, and deletions, were examined concerning different cancer types. Also, OS and DFS have been analyzed between the unaltered groups and the altered groups.

The association between USP22 expression and immune‐related variables, such as immune cell infiltration, immunoinhibitory and immunostimulatory elements, as well as HLA molecules, was examined through TISIDB. TISIDB examines the relationship between immune characteristics and gene expression in a range of cancer types by combining information from TCGA and other immune‐related datasets. A *p* value of < 0.05 is considered significant, and the system uses Pearson correlation analysis.

To analyze DNA methylation using OncoDB, we accessed the database at https://oncodb.org/ and navigated to the “methylation analysis” module. Methylation data, including beta values representing CpG site methylation levels, were extracted for both tumor and normal tissue [15]. The SMART website offers data on DNA methylation locations and CpG‐aggregated methylation [16, 17].

Protein–Protein network of the USP22 gene was obtained using STRING (version 12.0) (https://string‐db.org/). The six important genes were examined using the “Correlation Analysis” module of GEPIA2, which carried out Pearson correlation analysis. Scatter plots were generated using GEPIA2 to validate the “Correlation Analysis” and a heatmap displaying correlations and related *p* values generated using the “Gene Corr” module of TIMER2.0.

## 3 Results

### 3.1 Pan‐Cancer Analysis of the USP22 Gene Expression With Cancer Stages

**FIGURE 1:**
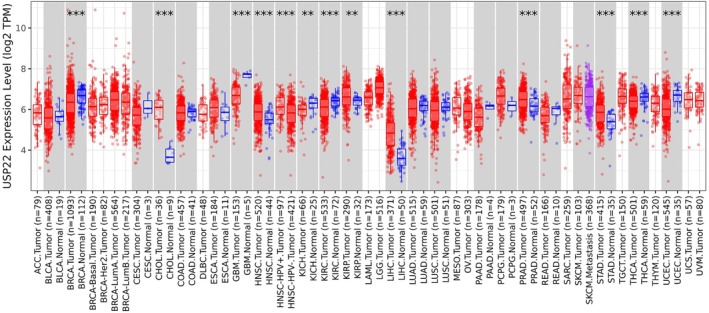
The expression of USP22 in various malignancies and the corresponding normal tissues was obtained using Timer 2.0. ACC, adrenocortical carcinoma; BLCA, bladder urothelial carcinoma; BRCA, breast invasive carcinoma; COAD, colon adenocarcinoma; DLBC, lymphoid neoplasm diffuse large B‐cell lymphoma; ESCA, esophageal carcinoma; GBM, glioblastoma multiforme; HNSC, head and neck squamous cell carcinoma; KICH, kidney chromophobe; KIRC, kidney renal clear cell carcinoma; KIRP, kidney renal papillary cell carcinoma; LAML, acute myeloid leukemia; LGG, brain lower grade glioma; LIHC, liver hepatocellular carcinoma; LUAD, lung adenocarcinoma; LUSC, lung squamous cell carcinoma; OV, ovarian serous cystadenocarcinoma; PAAD, pancreatic adenocarcinoma; PRAD, prostate adenocarcinoma; READ, rectum adenocarcinoma; SKCM, skin cutaneous melanoma; STAD, stomach adenocarcinoma; TGCT, testicular germ cell tumors; THCA, thyroid carcinoma; THYM, thymoma; UCEC, uterine corpus endometrial carcinoma; UCS, uterine carcinosarcoma. The number of stars represents the statistical significance as determined by the Wilcoxon test (**p < 0.01, and ***p < 0.001).

To explore the differential gene expression of interest between tumor tissues and adjacent normal tissues across all TCGA tumors, Timer 2.0 was employed. We found that USP22 expression was significantly higher in CHOL, HNSC, HNSC‐HPV+, KIRP, LIHC, PRAD, and STAD cancers (*p* < 0.001), and significantly lower in GBM, KICH, KIRH, THCA, BRCA, and UCEC (*p* < 0.001) (Figure 1). These 13 cancers were selected for further investigation. Further analysis of USP22 protein expression levels in several cancer types was conducted using the UALCAN web server with CPTAC and HPA databases. USP22 protein level was elevated significantly in breast cancer, lung cancer, head and neck cancer (HNC), clear cell RCC, and liver cancer compared to normal samples (*p* < 0.0001) (Figure S1a). Results of IHC testing for a number of cancer types demonstrate that malignant cells exhibited moderate to high nuclear staining, often in conjunction with cytoplasmic positivity. At the same time, hepatocellular carcinomas were generally weakly stained or negative (Figure S1b).

**FIGURE 2:**
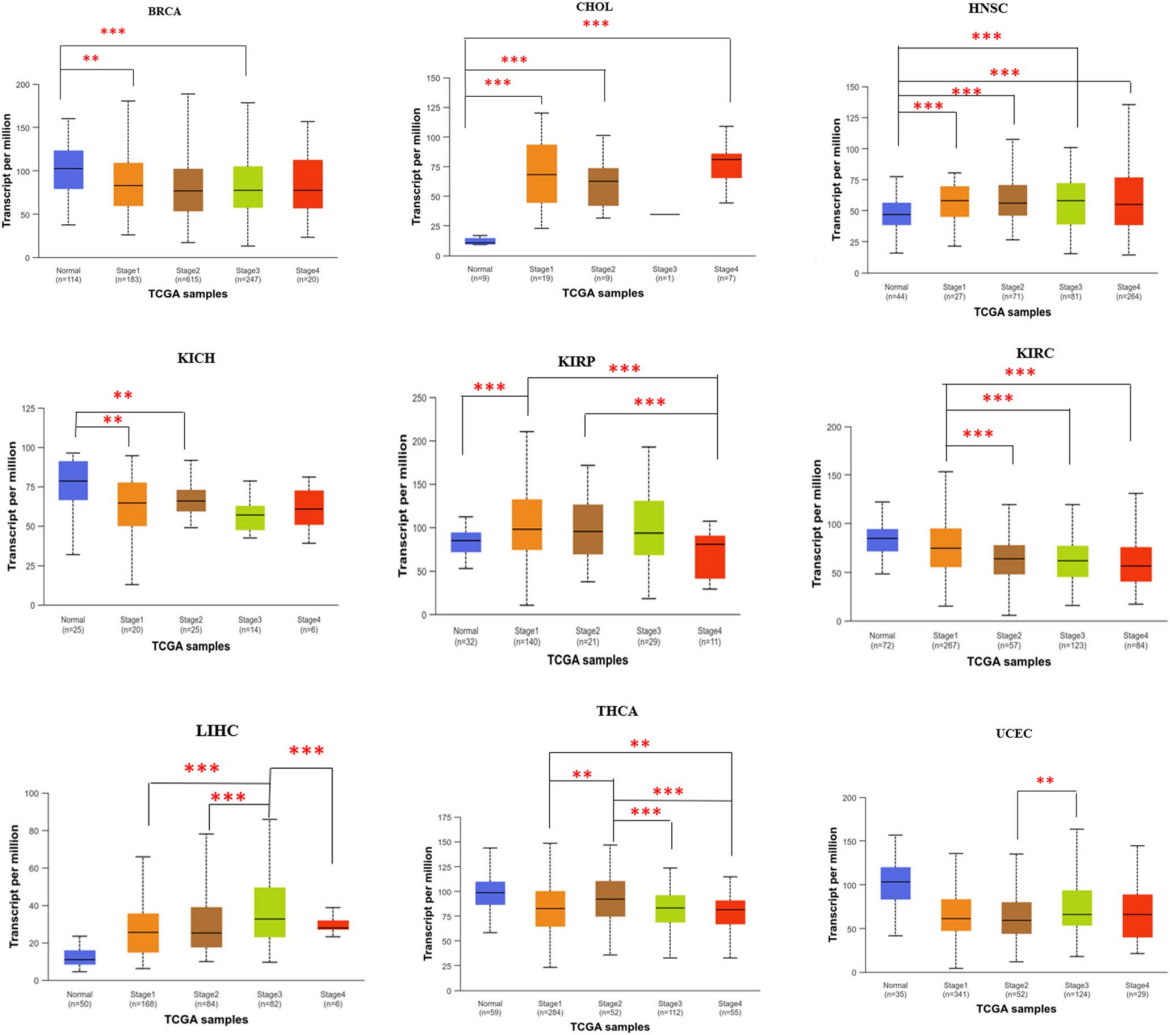
Using the UALCAN web server, USP22 expression level was significantly aligned with various stages of cancers, utilizing TCGA data. Differences in the expression across the stage were evaluated using one‐way ANOVA as implemented in the UALCAN platform; statistical significance was annotated by stars (**p < 0.01, and ***p < 0.001).

Further, we have analyzed the expression level of USP22 across different pathological stages of cancer using the UALCAN web server, an online platform for gene expression based on TCGA data. Among various cancer types, only those showing significant differential gene expression in TIMER 2.0 are shown here. In BRCA, KIRC, and THCA, USP22 expression was downregulated both in early (I/II) and advanced (III/IV) stages compared to normal, while in KICH, significant downregulation was observed in the early stage compared to normal (Figure 2, Table S1, *p* < 0.05). Upregulation of USP22 was seen in advanced cancer stages of CHOL, HNSC, LIHC, and UCEC compared to early and normal tissue (Figure 2, Table S1, *p* < 0.05). Stage‐wise expression analysis was carried out for other cancer types, which did not show significant differential expression, and the corresponding figures are provided in the Supporting Information. In the later stages of COAD and ESCA, upregulation was noted compared to both normal and early stages, whereas downregulation was observed in the advanced stages of LUSC, PAAD, and READ in comparison to the early stages (*p* < 0.05; Figure S2, Table S1).

In addition to pathological cancer stages, we also analyzed the expression of USP22 levels regarding tumor grade, histological subtypes, and TP53 mutation subtype using the UALCAN server. Focusing on various types of cancer, particularly brain cancer, LGG, we found that within the histological subtypes, oligodendroglioma shows greater USP22 expression than the other two subtypes, with a significant difference (*p* < 0.001; Figure S3, Table S1). Examining the TP53 mutant status in GBM indicates that USP22 expression is significantly elevated in normal tissue when compared to TP53‐mutated and TP53 nonmutant GBM samples, implying its downregulation in GBM (Figure S5, Table S1). In breast cancers, BRCA, the histological subtypes showed that USP22 was downregulated in infiltrating ductal carcinoma (IDC) compared to normal tissue, with a significance of *p* < 0.001. At the same time, no significant difference was observed between other subtypes. USP22 expression was significantly higher in normal breast cancer tissue compared to BRCA tumor samples. Nonetheless, no statistically significant differences were found between TP53‐mutant tumors and those that are TP53 nonmutant, suggesting that TP53 mutation status does not significantly influence USP22 expression in BRCA (Figure S5, Table S1). In cervical cancer CESC, analysis of tumor grade showed USP22 expression was considerably elevated in Grade 3 tumors compared to Grade 1 tumors (*p* < 0.01; Figure S4, Table S1). In the analysis of their histological subtypes in relation to the normal sample, all subtypes demonstrated a reduced level of USP22 expression. Mucinous carcinoma displays a higher median expression when compared to squamous cell carcinoma and endocervical carcinoma, with a significance level of approximately (*p* < 0.01; Figure S3, Table S1). Analysis of COAD histological subtypes in colon cancer showed that other subtypes had higher levels of USP22 expression than the normal sample, with mucinous adenocarcinoma and the normal sample differing significantly (Figure S3, Table S1). Analysis of the TP53 mutation status revealed that the nonmutant TP53 sample differed significantly from the normal sample (Figure S5, Table S1). In ESCA, USP22 expression in normal samples appears relatively low compared to tumor samples; squamous cell carcinoma exhibits higher median USP22 expression compared to adenocarcinoma (*p* < 0.01; Figure S3, Table S1). In HNSC, the expression level of USP22 increases progressively with tumor grade compared to normal tissue. Tumor Grade 4 shows the highest median expression, indicating a possible association between USP22 expression and tumor progression (*p* < 0.001; Figure S4, Table S1). Meanwhile, in the TP53 mutation status, the expression of USP22 in HNSC increased significantly in both the TP53 mutant stage and nonmutant stage in relation to the normal tissue (*p* < 0.001; Figure S5, Table 1). In renal cancers, KIRC, analyzing its tumor grade shows that USP22 expression was higher in early tumor grade samples compared to normal, while later tumor grade samples showed reduced expression compared to Grade 2 tumors, implying that USP22 may contribute to tumor initiation (*p* < 0.001; Figure S4, Table S1). KIRP, compared to normal tissue, USP22 expression was elevated in all cancerous subtypes, with significance noted between Type 2 PRCC versus unclassified PRCC and Type 1 PRCC versus Type 2 PRCC (*p* < 0.002; Figure S3, Table S1). When it comes to liver malignancies, LIHC, the normal group shows the lowest USP22 expression level, but USP22 expression was significantly higher in all malignant subtypes, with hepatocellular carcinoma and fibrolamellar carcinoma showing the most upregulation (*p* < 0.001; Figure S3, Table S1). Then their tumor grade was analyzed; both early tumor grade (1/2) and later tumor grade (3/4) showed considerably higher expression of USP22 than normal tissue (*p* < 0.001; Figure S4, Table S1). TP53 mutation status: The normal group has the lowest expression levels, while LIHC samples from the TP53‐mutant and TP53 nonmutant stages show significantly higher expression. Notably, the TP53 mutant group has slightly higher expression than the nonmutant stage, indicating that TP53 mutation is associated with the progression of liver cancer (*p* < 0.001; Figure S5, Table S1). According to LUAD, histological subtype analysis reveals that the normal sample has a moderate level of USP22 expression, while some subtypes, such as mucinous, clear cell, micropapillary, and mucinous carcinoma, have significantly higher levels of USP22 expression than the normal and other subtypes (*p* < 0.001; Figure S3, Table S1). USP22 expression is elevated in the TP53 mutant group than in the TP53 nonmutant group, according to TP53 mutation status analyses of LUAD. This suggests that USP22's presence in the TP53 mutation sample affects its expression in LUAD (*p* < 0.01; Figure S5, Table S1). According to skin cancer analysis of TP53 mutant status in SKCM, TP53 nonmutant status exhibits a higher level of USP22 expression than TP53 mutant status (Figure S5, Table S1). All malignant subtypes exhibit more significant (*p* < 0.001) overexpression of UPS22 in stomach cancer, STAD, as compared to the normal sample (Figure S3, Table S1). Grade 3 tumor shows higher expression of USP22 compared to Grade 1 tumor (*p* < 0.001, Figure S4, Table S1). In TGCT cancer, a significant difference in USP22 expression was observed between seminoma vs. Non‐seminoma subtypes (*p* < 0.001; Figure S3, Table S1). Histological subtype studies of THCA cancer reveal substantial differences (*p* < 0.05) between normal samples and specific subtypes such as follicular, tall, and classical (Figure S3, Table S1). When UCEC cancer histological subtypes are analyzed, it is found that USP22 expression in the subtypes is statistically significantly reduced compared to the normal sample, with notable variation in endometrioid and serous (*p* < 0.001; Figure S3, Table S1). Comparing the TP53 mutation and non‐mutation samples to the normal sample, USP22 expression was lower (Figure S5, Table S1). In PAAD, tumor grade states that a significant reduction in USP22 expression occurs between early tumor grade (1/2) and later grade [3] (*p* < 0.05; Figure S4, Table S1). TP53 mutant status states that TP53 nonmutant expression was higher than TP53 mutant samples, and compared to the normal sample, both TP53 mutant and nonmutant samples have reduced USP22 expression (*p* < 0.001; Figure S5, Table S1). In PRAD, normal prostate samples exhibit moderate USP22 expression levels, while both TP53 mutant PRAD and TP53‐nonmutant PRAD display significantly higher USP22 expression compared to normal tissue (*p* < 0.001; Figure S5, Table S1).

**TABLE 1:** USP22 gene expression with survival probability with different cancers according to the TCGA database using the UALCAN web server.

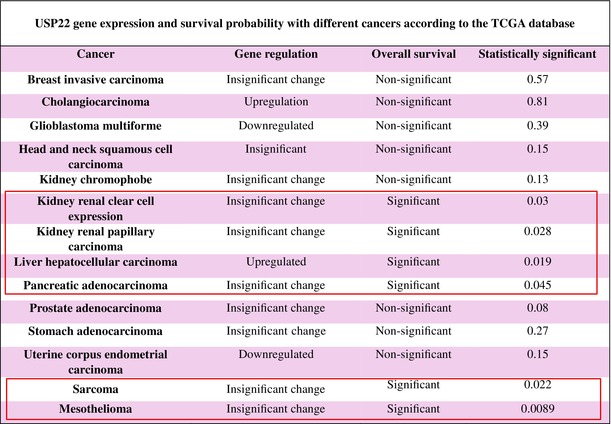

*Note:* Coloured box indicate statistical significance result (*p *< 0.05).

### 3.2 Prognostic Analysis of USP22

**FIGURE 3:**
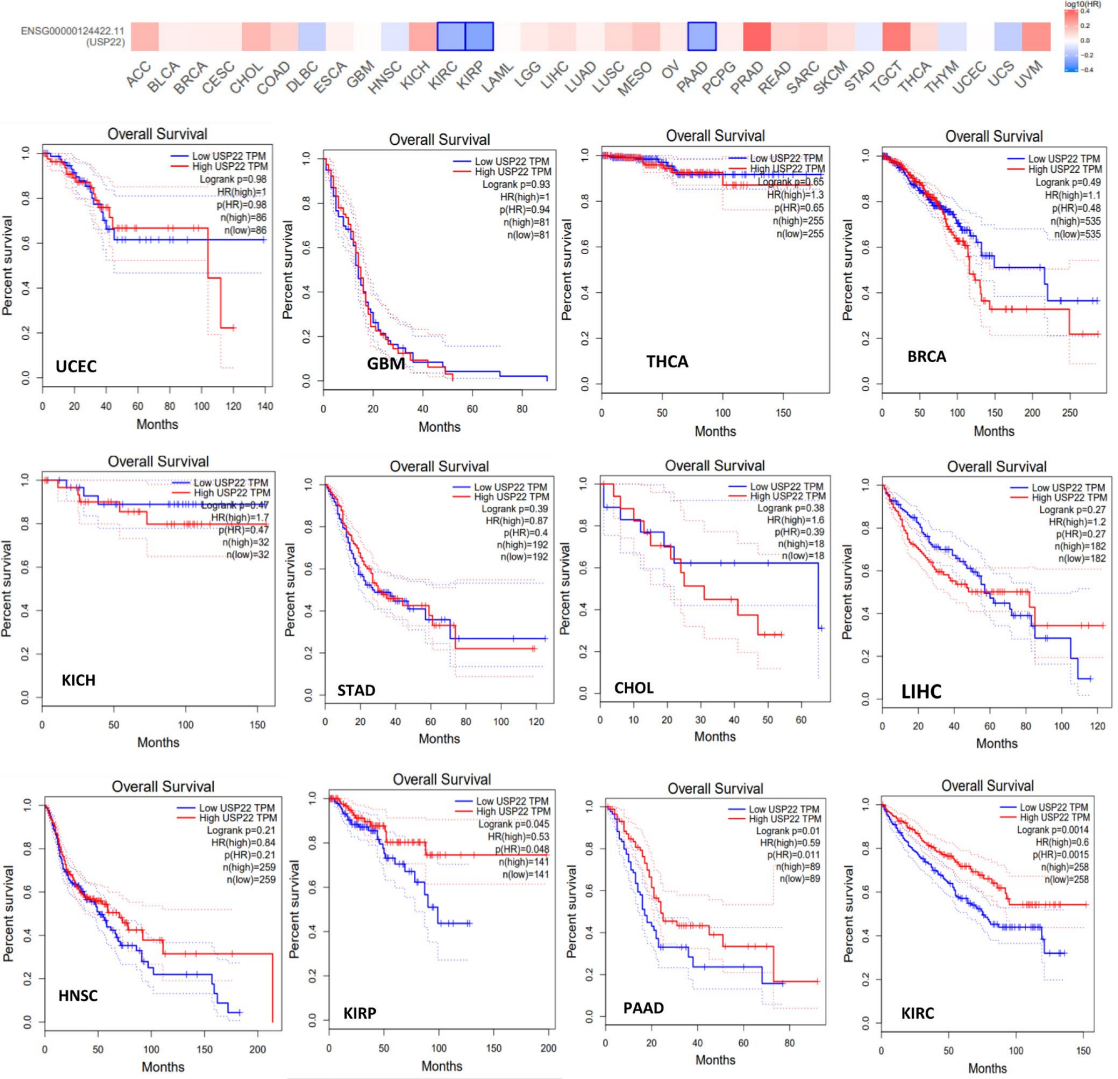
Overall survival analysis was conducted using GEPIA2, an online platform for expression data analysis based on TCGA and GTEx datasets, using Kaplan–Meier plots. High expression is represented by red lines, and blue lines represent low expression levels. Hazard ratios were estimated with a Cox model and 95% confidence intervals.

**FIGURE 4:**
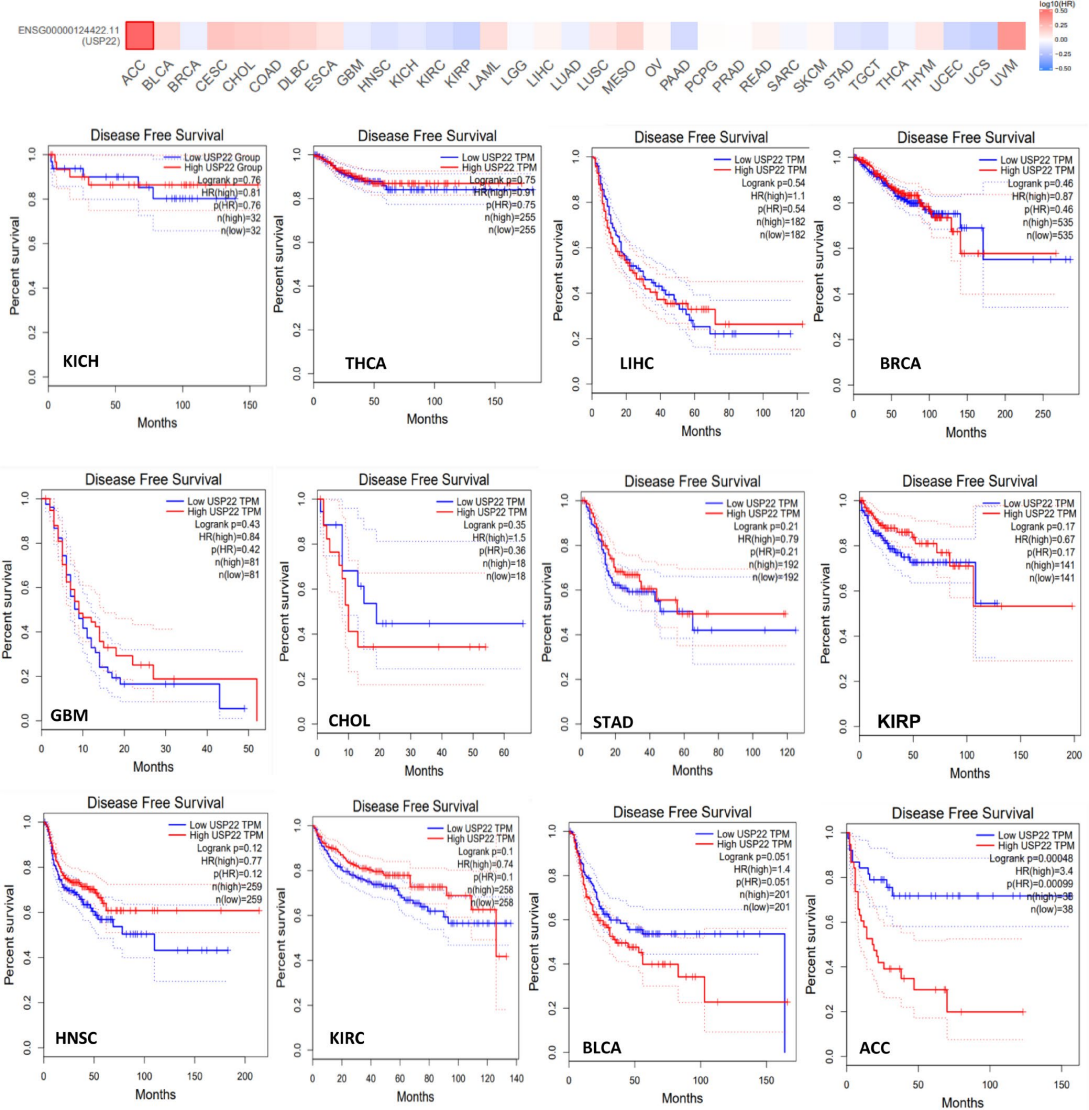
Disease‐free survival (DFS) analysis was performed using GEPIA2 integrated expression data from TCGA and GTEx, using Kaplan–Meier plots. Hazard ratios were calculated using the Cox model with 95% confidence intervals.

By using the UALCAN web server designed for pan‐cancer analysis, which enables users to scan gene classes related to particular biological pathways or functions, we systematically explored deubiquitinase‐related genes (USP22) and correlated them with the cancer‐specific data. The survival probability for cancer associated with USP22 was analyzed, and data revealed that the survival probability fell with time in patients with elevated expression levels of USP22 compared to lower expression levels in KIRC, KIRP, LIHC, PAAD, SARC, and MESO (*p* < 0.05). For the malignancies BRCA, GBM, HNSC, KICH, PRAD, STAD, and UCEC, USP22 expression might not be a relevant prognostic indicator (Figure S6, Table 1). Increased levels of USP22 expression were significantly linked to reduced OS rates in patients with KIRC, KIRP, and PAAD (*p* < 0.05) (Figure 3). Data on the DFS time in the TCGA tumor types indicated that greater levels of USP22 expression were associated with a poorer prognosis compared to lower levels of expression. DFS prognosis showed substantial alteration for tumor ACC and BLCA (*p* < 0.05) (Figure 4). According to our studies, there was no notable correlation between USP22 expression and OS or DFS in certain cancers, BRCA, GBM, HNSC, KICH, PRAD, STAD, and UCEC. These findings indicate that USP22 overexpression in the aforementioned malignancies causes worse clinical outcomes.

**FIGURE 5:**
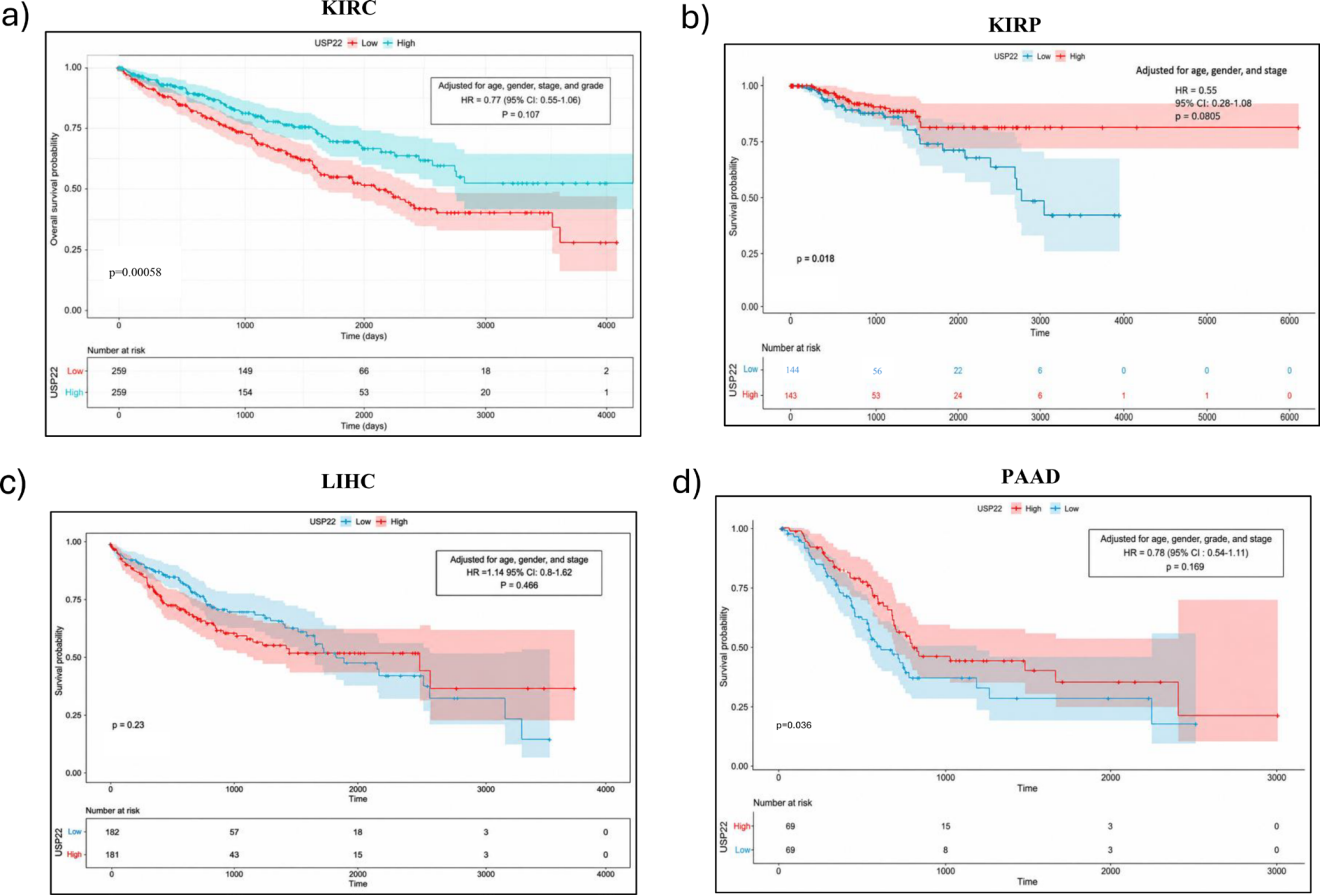
Kaplan–Meier survival analysis of USP22 expression in (a) KIRC, (b) KIRP, (c) LIHC, and (d) PAAD. HRs with 95% CIs were calculated using multivariate Cox regression adjusted for age, gender, tumor grade, and clinical stage. The log‐rank test was used to determine statistical significance.

A multivariate Cox regression analysis was done to estimate HRs with 95% CIs in malignancies KIRC, KIRP, LIHC, and PAAD, where USP22 was significant in univariate analysis, controlling for age, gender, grade, and cancer stage. In KIRC, multivariate Cox analysis revealed that increased USP22 expression was associated with better survival (HR = 0.77, *p* = 0.107). Age was substantially related to a lower survival rate (HR = 1.03, *p* < 0.001), whereas gender had no significant effect. Patients in Stages III and IV had considerably worse survival rates than those in Stage I (HR =2.01, *p* = 0.001; HR = 5.24, *p* < 0.001). High tumor grade was related to shorter survival than low grade (HR = 1.57, *p* < 0.001) (Table 2, Figure 5a, Figure S8). In KIRP, high expression was significantly associated with improved survival probability (HR = 0.54, *p* = 0.008). Poorer survival was significantly associated with advanced tumor Stages III and IV (HR = 4.98, *p* = 0.0023; HR = 13.46, *p* < 0.001) (Table 2, Figure 5b, Figure S9). LIHC showed no significant relationship between USP22 expression and OS (HR = 1.14, 95% CI: 0.80–1.62, *p* = 0.466). Individuals in the later stage had a significantly higher risk of death (HR = 2.44, 95% CI: 1.72–3.47, *p* < 0.0001). Gender and age had no effects on survival (Table 2, Figure 5c, Figure S10). Analyzing PAAD, USP22 expression was linked to improved survival; this impact was not significant when clinical variables were taken into account. Survival was not substantially correlated with tumor stage, grade, or gender (Table 2, Figure 5d, Figure S11).

**TABLE 2:** Multivariate Cox regression analysis evaluating the prognostic significance of USP22 expression, adjusted for age, gender, tumor grade, and clinical stage.

Cancer	Variable	HR	95% CI	*p*
KIRC	USP22	0.77	0.56–1.06	**0.107**
	Age	1.03	1.02–1.05	**< 0.001**
	Gender (male)	0.92	0.67–1.26	0.625
	Stage II vs. I	1.16	0.62–2.17	0.638
	Stage III vs. I	2.01	1.31–3.07	**0.001**
	Stage IV vs. I	5.24	3.46–7.94	**< 0.001**
	Grade (high vs. low)	1.57	1.09–2.27	**< 0.001**
KIRP	USP22	0.54	0.279–1.076	**0.008**
	Age	0.99	0.97–1.027	0.92
	Gender (male)	0.625	0.313	0.181
	Stage II vs. I	0.98	0.27–3.521	0.98
	Stage III vs. I	4.98	1.219–14.460	**0.023**
	Stage IV vs. I	13.462	3.580–50.622	**< 0.001**
LIHC	USP22	1.14	0.80–1.62	0.466
	Age	1.01	1–1.03	0.11
	Gender (male)	0.92	0.64–1.32	0.655
	Stage (late vs. early)	2.44	1.72–3.47	**< 0.0001**
PAAD	USP22	0.78	0.54–1.11	0.169
	Age	1.02	1.00–1.04	0.059
	Gender (male)	0.77	0.50–1.16	0.213
	Stage II vs. I	1.73	0.77–3.89	0.183
	Stage III vs. I	0.69	0.08–5.81	0.731
	Stage IV vs. I	1.42	0.28–7.09	0.668
	Grade II vs. I	1.48	0.76–2.89	0.2540
	Grade III vs. I	1.78	0.87–3.65	0.1160
	Grade IV vs. I	0.70	0.08–6.30	0.7470

*Note:* Bold values indicate statistical significane (*p* < 0.05).

### 3.3 USP22 Gene Alteration in Various Cancers

**FIGURE 6:**
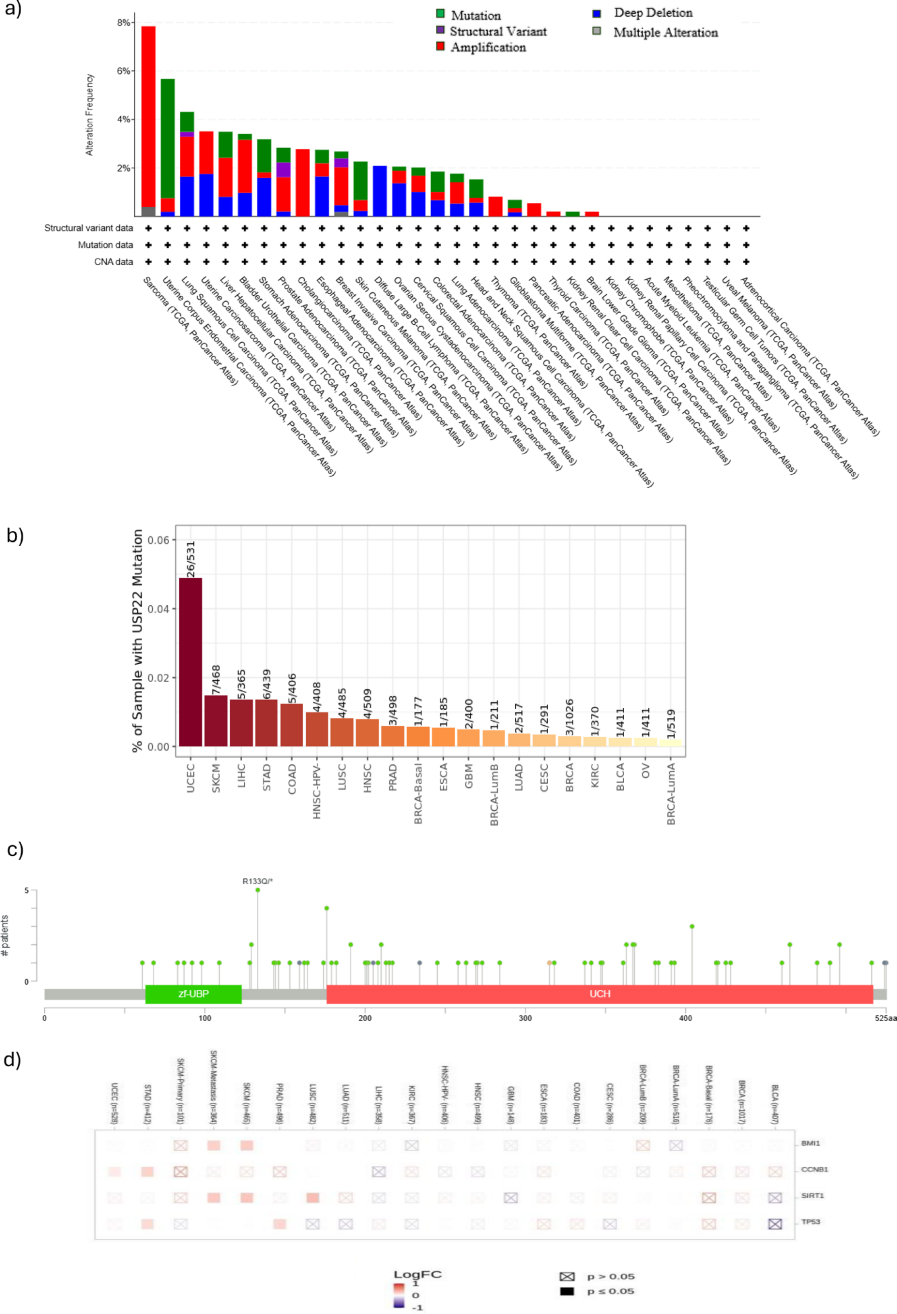
Gene alteration of USP22 in various cancers. (a) Alteration frequency with mutation types. (b) Percentage of samples with USP22 mutation. (c) Alteration frequency with mutation sites. (d) Differential gene expression between the mutation statuses.

The TCGA cohorts datasets were utilized to investigate the genetic alterations in USP22. The cBioportal tool is used for various tumor types. As shown in Figure 6a, Table S2, USP22 has the highest alteration frequency (~8%) in sarcoma with “Amplification” as the main type. Uterine corpus endometrial carcinoma is nearly as high as sarcoma, primarily driven by mutation and amplification, with a frequency of ~6%. Other common malignancies include BLCA, LIHC, uterine carcinoma, and LUAD, with an alteration frequency of ~5%, ~4%, ~4%, and ~3%. A variety of mutations, amplifications, and deletions cause cancers such as BRCA and PRAD. Amplifications, deletions, and mutations all contribute to LUSC in a balanced way. The lowest alteration frequency was observed with THCA, kidney renal clear cell carcinoma, and brain lower‐grade glioma. Figure 6b shows the percentage of samples with USP22 mutation across various cancer types, and missense mutations are the most predominant type. Comparing this to other cancer kinds, 26 out of 531 samples (~4.89%) with UCEC have USP22 mutations, and 7 out of 468 samples (~1.5%) with SKCM have USP22 mutations. The rates of mutation in LIHC, STAD, and COAD vary from approximately 1% to 1.3%. Lowest mutation prevalence: Very low mutation rates (~0.1% or less) are found in many malignancies, including BLCA, OV, and breast cancer subtypes (BRCA‐LumB and BRCA‐LumA). Figure 6c shows the frequency of alterations associated with various mutation types. Zf‐UBP is a zinc finger domain important for association with the other component of the SAGA complex, and the UCH ubiquitin C‐terminal hydrolase domain is required for enzymatic activity.

Mutations are scattered over the entire length of the protein, although clusters arise in certain areas. A prominent cluster around the Zf‐UBP domain (e.g., residues 50–150 aa). Further clustering in the UCH domain (residues about 200–400 aa). The R133Q mutation is indicated, showing its recurring nature occurs in up to 5 patients; this modification was most prevalent in uterine corpus endometrial cancer and COAD.

According to Figure 6d, the USP22 mutation, the heatmap shows the log fold change (LogFC) in expression for particular genes (BMI1, CCNB1, SIRT1, and TP53) across different cancer types, and BMI1 is upregulated in SKCM and BRCA cancer. CCNB1 upregulates in STAD and UCEC. Sirtuin 1 (SIRT1) is highly upregulated in LUSC, SKCM, and SKCM metastasis. TP53 is upregulated in PRAD and STAD. A substantial association was found between OS, disease‐specific survival, and USP22 alteration (*p* value < 0.05). Additionally, there was a strong association (*p* value < 0.05) between poor prognosis and overall patient survival (Figure S7a,b, Tables S3–S6).

### 3.4 USP22 Expression Patterns in Cancer‐Associated Immune Signatures

**FIGURE 7:**
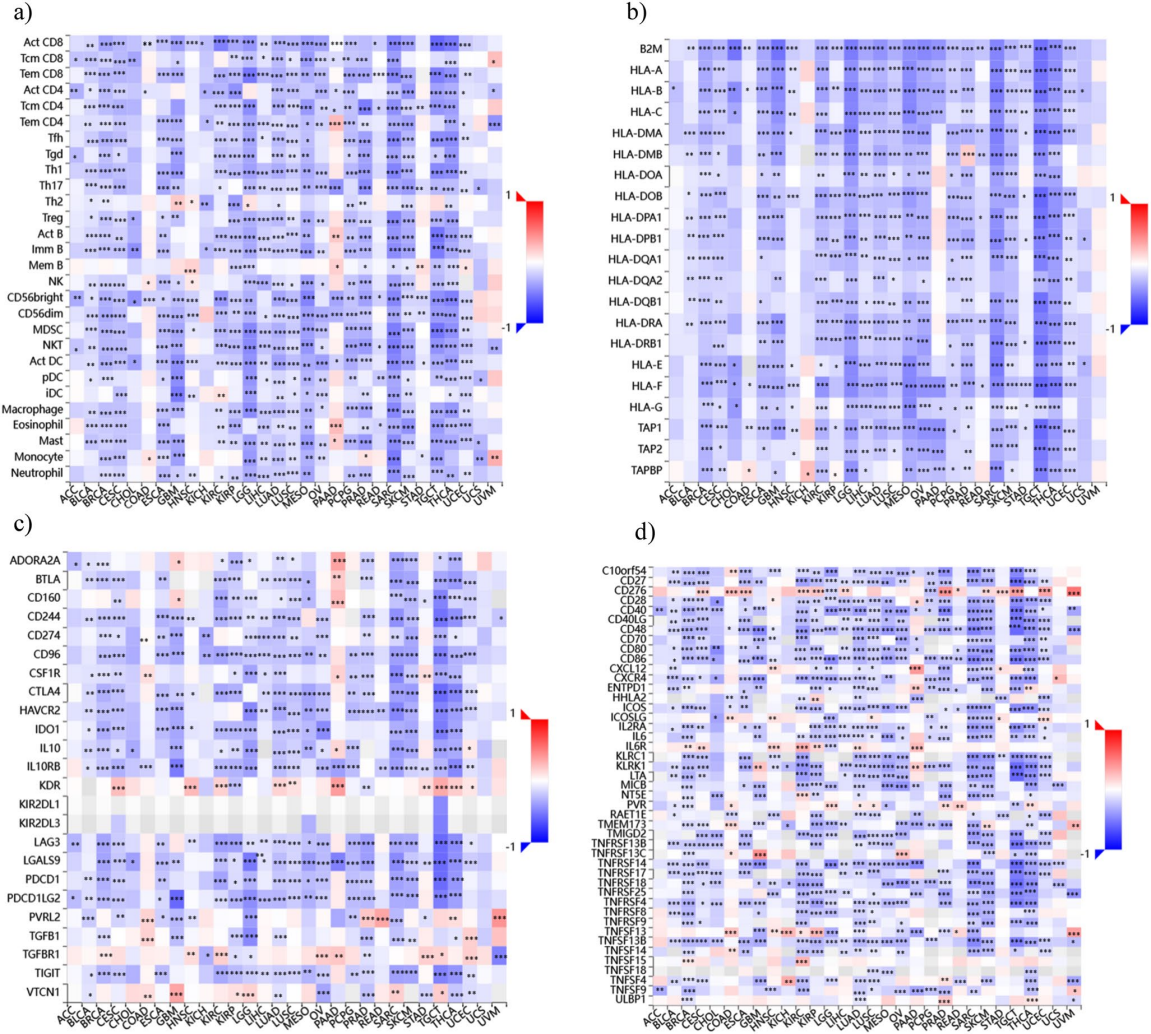
The relationship between USP22 expression and immune‐related signatures. USP22 has a strong correlation with (a) immune cells, (b) HLA molecules, (c) immunostimulators, and (d) immunoinhibitors across human cancer.

The heatmap (Figure 6) illustrates the different expressions of USP22 across various immune cell types and provides insights into its role in cancer immunology. According to the USP22 expression in immune cells, it shows a positive correlation with Tem CD4 cells, Th2, MemB, Eosinophil, monocyte, and mast cells in GBM, HNSC, PAAD, STAD, and UVM cancers; however, there is a negative correlation between USP22 expression and immune cell types including activated CD8 T cells, regulatory T cells (Treg), T helper cells (Th1 and Th17), B cells, NK cells, dendritic cells, macrophages, and neutrophils, suggesting that USP22 may facilitate tumor immune evasion by promoting an immunosuppressive tumor microenvironment (Figure 7a). Additionally, the majority of HLA Class I and Class II molecules demonstrated a negative association with USP22 expression across numerous malignancies, and nearly all HLA molecules had a substantial negative correlation with USP22 expression (Figure 7b). USP22 expression with immunostimulators revealed that ADORA2A, BTLA, CD160, KDR, CSF1R, PVRL2, TGFBR1, and VTCN1 showed strong positive correlation in BRCA, GBM, LGG, PAAD, KIRC, READ, TGCT, UCEC, SARC, and COAD cancers, stating multiple immunosuppressive processes can be coordinated by USP22 in malignancies (Figure 7c). In regard to immune inhibitors, in PAAD, GBM, OV, KIRC, TGCT, THCA, UCEC, and KIRP malignancies, USP22 expression is highly correlated with CD276, CXCL12, IL6R, PVR, TNFRSF13C, TNFSF13B, and TNFSF4 (Figure 7d).

### 3.5 USP22 DNA Methylation Analysis in Cancer

**FIGURE 8:**
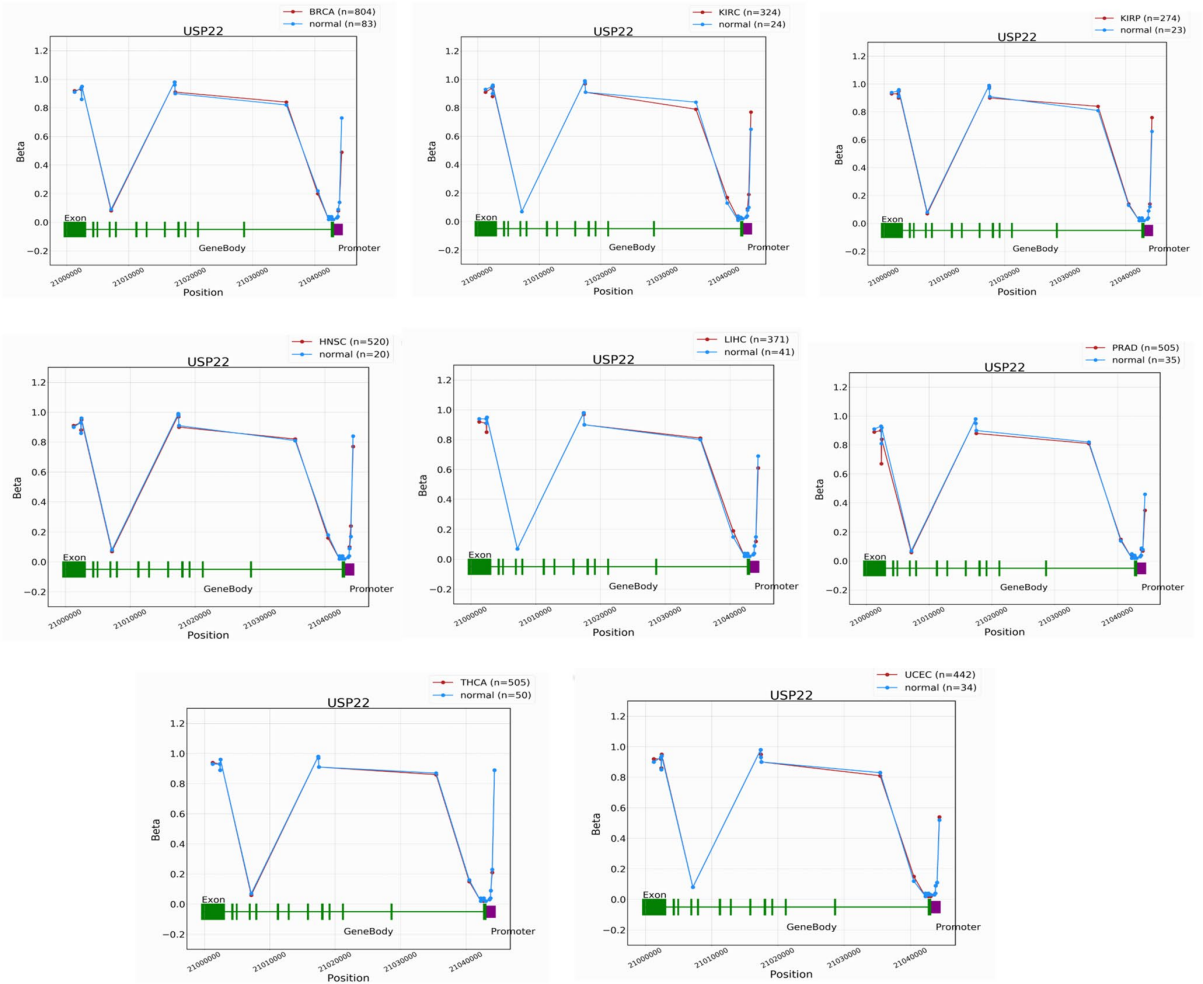
USP22 DNA methylation analysis in cancers using the OncoDB database. It allows users to input a gene of interest and visualize its methylation status across different cancer types.

**FIGURE 9:**
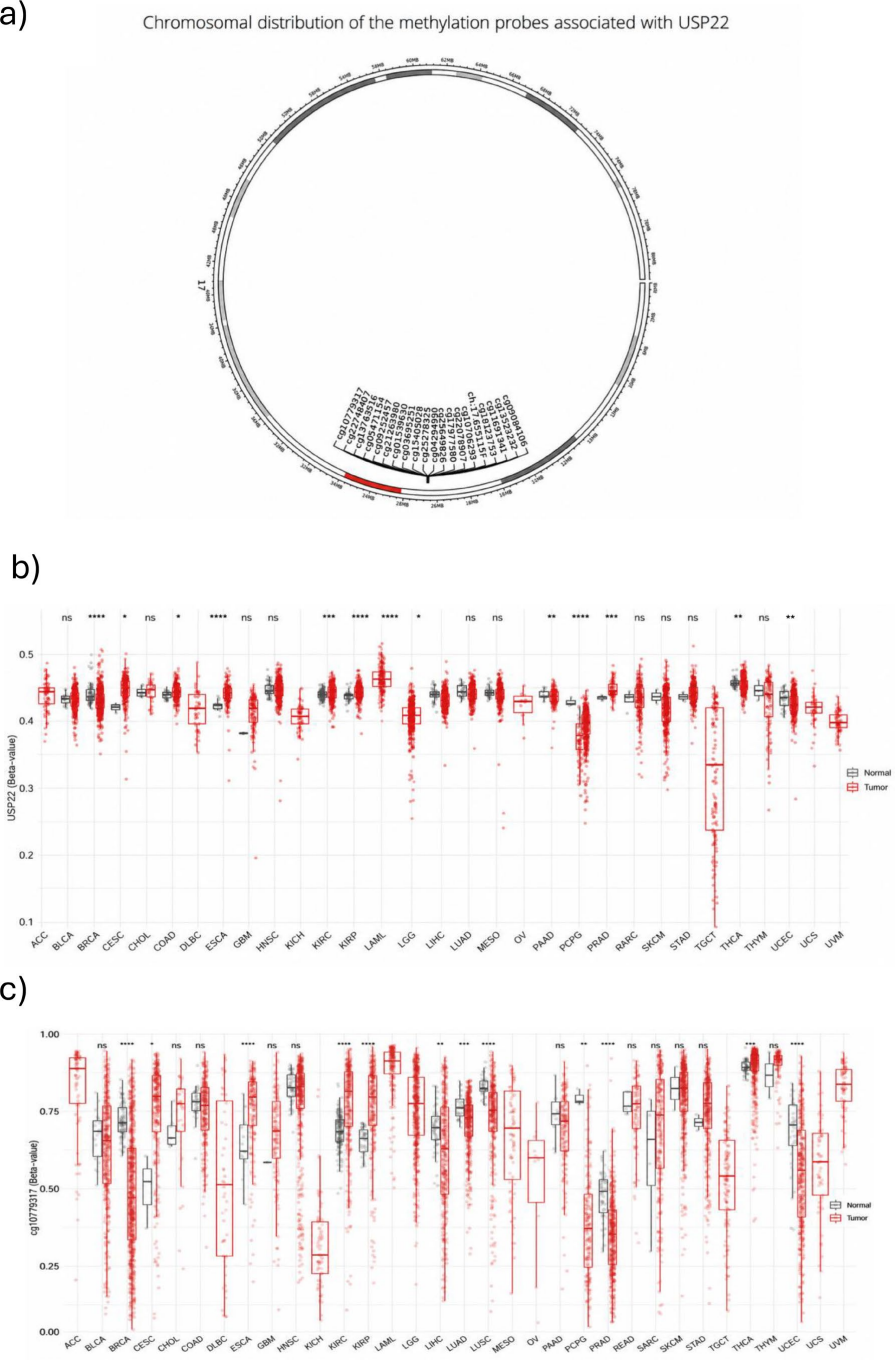
Methylation level of USP22 in various tumors. (a) Chromosomal location of the methylation probes associated with USP22. (b) Aggregated methylation levels across all CpG sites in USP22 in human cancers with SMART. (c) DNA methylation *β* values at a specific CpG probe (cg10779317) in the S shore region of USP22 expression in tumor (red) versus normal (black) tissues across various cancer types, with statistical significance assessed using the Wilcoxon test indicated by the number of stars (*p < 0.05, **p < 0.01, ***p < 0.001, and ****p < 0.0001).

We have also analyzed the USP22 DNA methylation in BRCA, KIRC, KIRP, HNSC, LIHC, PRAD, THCA, and UCEC cancers. The DNA methylation analysis of USP22 in all cancers reveals significant epigenetic alterations. The methylation beta value, 0 to 1, indicates the state of unmethylation to hypermethylation in all mentioned cancers; a clear pattern of hypomethylation in the promoter region and hypermethylation in the gene body is seen compared to normal tissues. The promoter region shows a decrease in methylation, suggesting USP22 overexpression in all malignancies. Meanwhile, hypermethylation in the gene body indicates active transcription regulation, which contributes to tumor progression by modulating ubiquitination and gene regulation (Figure 8). Additionally, we investigated USP22 methylation in a number of cancer studies, comparing methylation levels in cancerous and normal tissue using the SMART database, an interactive online application for thorough DNA methylation analysis and visualization. The chromosome‐wide distribution of methylation probes connected to the USP22 gene is shown in Figure 9a. According to the CpG‐aggregated methylation study, USP22 is significantly hypomethylated in tumor samples relative to normal tissue in CESC, THCA, COAD, LUAD, BRCA, LIHC, PRAD, PCPG, READ, and UCEC. While KIRC, KIRP, and ESCA showed considerably greater CpG‐aggregated methylation levels of USP22 in tumor tissue as compared to normal controls (Figure 9b). Tumor samples have noticeably lower methylation levels at the cg10779317 probe than normal tissues in the majority of cancer types, including CESC, BRCA, LUAD, LUSC, LIHC, PCPG, PRAD, THCA, and UCEC. While ESCA, KIRC, and KIRP showed significantly higher methylation between tumor and normal tissue (Figure 9c).

**FIGURE 10:**
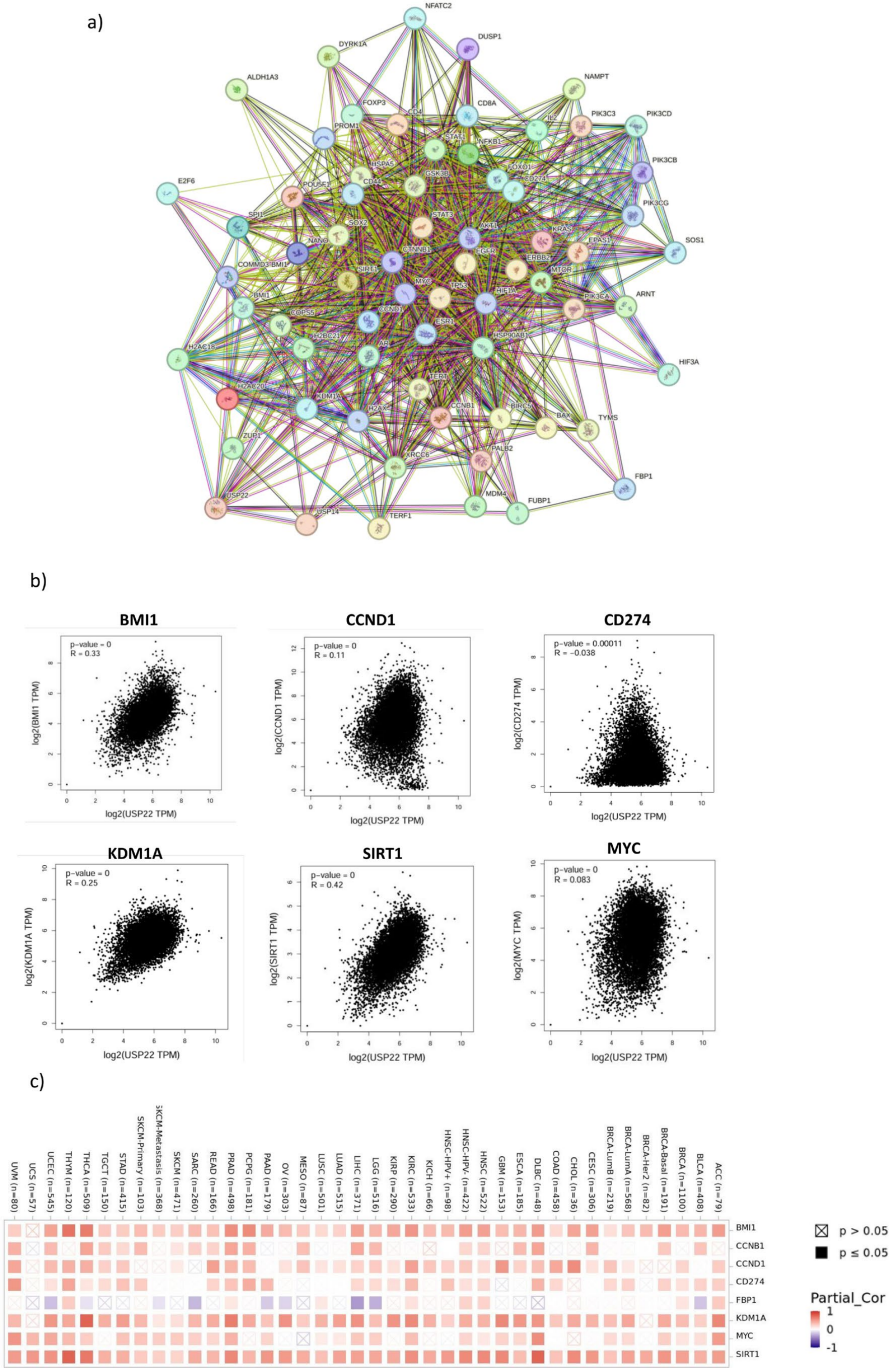
(a) The USP22‐binding proteins (PPI) utilizing the STRING tool. (b) The correlation between the expression of USP22 and the six co‐expressed genes (C‐Myc, KDM1A, SIRT1, BMI1, CCNB1, and CCND1). (c) The heatmap depicts the correlation between the USP22 and the six co‐expressed genes in multiple cancer types.

Figure 10a displays the findings of the network of protein–protein interactions (PPIs) of USP22. The top six proteins C‐Myc, KDM1A, SIRT1, BMI1, CCNB1, and CCND1 were directly connected to USP22. The strong positive correlation between SIRT1 (*R* = 0.42), BMI‐1 (*R* = 0.33), KDM1A (*R* = 0.25), CCND1 (*R* = 0.11), and weak correlations observed between CD274 (*R* = −0.038) and MYC (*R* = 0.03), along with USP22 expression (Figure 10b). Additionally, the heatmap analysis findings showed that those six genes and USP22 expression were positively correlated in all cancer types (Figure 10c).

## 4 Discussion

The USP22 belongs to the USP family and is often termed the “death from cancer” gene, which is critically involved in cancer progression and resistance to therapy [18]. Additionally, it is necessary for cell cycle progression; USP22 depletion causes G1 phase cell cycle arrest, and it regulates cell cycle progression [19]. Although USP22 has been implicated in various cancers, a pan‐cancer analysis has not been previously reported. This study addresses that gap by systematically evaluating its epigenetic status across multiple tumor types. Our comprehensive pan‐cancer analysis of USP22 expression across numerous tumor types showed notable variations in expression patterns observed between tumor and normal tissues, as well as across different cancer stages, histological subtypes, TP53 mutation status, and DNA methylation status. The differential gene expression analysis showed USP22 to be significantly expressed in multiple cancers, including BRCA, CHOL, HNSC, HNSC‐HPV+, KIRH, KIRP, KICH, GBM, LIHC, PRAD, STAD, THCA, and UCEC, which aligns with the previous reports where USP22 is said to be overexpressed in at least 14 solid tumors, including glioma, breast cancer, thyroid cancer, lung cancer, colorectal cancer, prostate cancer, and it has been linked with poor prognosis, supporting its role in proto‐oncogene and its therapy resistance [9, 20].

Along with the USP22 overexpression in the pathological stages of the TCGA sample, it showed both upregulation and downregulation in the histological subtypes, TP53 mutant stages, and tumor grade in numerous cancers compared to the control. When USP22 expression was analyzed in four individuals, it was shown that recurrent colorectal cancer had greater USP22 levels than primary colorectal cancer [21]. Overexpression of the USP22 protein was discovered in 45% of NSCLC tissue and was associated with patients' larger tumors and lymph node metastases [22, 23, 24]. It is yet unknown why some cancer types (BRCA, KIRC, PAAD, and UECE) exhibit downregulation of USP22; this might be due to context‐dependent regulatory mechanisms that need more research. A lack of USP22 causes chromatin compaction abnormalities and hinders the clearance of H2Bub1. Additionally, USP22 is a new CIN gene, suggesting that tumors' deletions of USP22 may trigger CIN and aid in oncogenesis [25].

The protein level provided additional confirmation of this transcriptional upregulation, shown by the CPTAC data, which shows that tumor samples from several malignancies have higher USP22 expression than normal tissue in multiple cancers. The samples of liver cancer were weakly stained in many samples. Numerous other investigations have verified that USP22 is overexpressed at the protein level in various cancer types and that this is associated with poor patient survival [26, 27, 28]. This concordance between transcriptomic and proteomic levels strengthens the validity of USP22 as a cancer‐associated molecule.

Genetic alterations analysis was used to evaluate a gene's oncogenic functions in certain cancer types. Our data indicate that Sarcoma and UCEC have the highest genetic alteration frequency. The genetic mutations were prevalent in UCEC and SKCM, followed by LIHC, STAD, and colon cancer, which cumulatively indicate that the USP22 mutation influences cancer progression [29]. The deep deletion was a dominant alteration in LUSC, UCS, ESCA, and DLBCL. In addition, the OS and DFS showed that the USP22‐altered groups have a poor survival rate compared to the unaltered group. Since USP22 has been reported as a potential prognostic biomarker in various cancers [20], we assessed its association with patient outcomes. The study found a strong correlation between higher USP22 expression and patients' poor prognosis, reduced survival probability, and OS in SARC, PAAD, LIHC, and MESO cancers, which correlated with the previous reports where USP22 was stated to be an independent prognostic parameter in HCC [30], breast cancer [31], papillary THCA [32], pancreatic cancer [33], and OSCC [26]. In addition, individuals with ACC, BLCA, and CHOL have a poor DFS prognosis when USP22 expression is increased [28, 34]. Additionally, prior research using KM analysis has demonstrated that patients with non‐small cell lung cancer with high expression have a worse OS rate [35, 36]. A multivariate Cox regression analysis revealed that increased USP22 expression is linked to improved OS in KIRC and KIRP, despite the fact that USP22 increases tumor cell survival through surviving stability [37]. This may be explained by the observation that USP22 expression is higher in early‐grade tumors and decreases in advanced stages in KIRC and KIRP. While in LIHC, advanced tumor stages have shown the worst outcomes in patients. USP22 has also been linked to a CSC‐promoting role and is closely linked to tumor recurrence, metastasis, resistance to traditional treatments, and poor survival in a variety of cancer types.

In tumor‐promoting inflammation, USP22 is allegedly elevated, preventing immune destruction [19, 38]. Our research observed that elevated levels of USP22 in tumors are associated with an immunosuppressive microenvironment. This association correlates with reduced infiltration of cytotoxic and helper immune cells, downregulation of HLA molecules necessary for antigen presentation, and increased levels of various immunosuppressive and checkpoint molecules. A prior study found that pancreatic tumor cells with USP22 deleted had a better response to the combination immunotherapy because myeloid cell infiltration was decreased, and T cell and NK cell infiltration increased [39]. For maintaining immune response control and homeostasis, FOXP3 regulatory T cells are essential, but they also pose a significant challenge to antitumor immunity. FOXP3 expression is positively regulated by USP22. Using CRISPR technology to knock down USP22 in Treg cells declines Foxp3 protein expression and suppresses tumor growth in numerous scenarios [40]. Knocking down USP22 leads to decreased T‐cell‐dependent tumor metastasis, increased tumor immunogenicity, lymphatic invasion, and natural killer cell activity [39]. USP22 reduction demonstrated the intricate roles of the USP22‐CD274 axis in the effectiveness of cancer treatment by improving CDDP‐based chemotherapy and increasing the therapeutic efficiency of CD274‐targeted immunotherapy [41]. The function of USP22 in the immunological microenvironment might develop into a new area of interest. There isn't much evidence available right now, and further research is required to fully understand the systems involved.

DNA methylation is a key component of epigenetics, and its dysregulation is associated with the development of carcinogenesis [42]. DNA methylation has a key role in the regulatory tumor‐related gene expression in patients with the condition [43]. According to the study, the promoter region of USP22 showed a lower methylation level, which facilitates increased gene expression, which is consistent with the observed overexpression of USP22 in multiple cancers [19, 29, 44]. Hypomethylation can occur early in carcinogenesis and is typically found in benign hyperplasia. Methylation loss increases with tumor development, with metastatic lesions exhibiting higher levels of demethylation than initial tumors [45]. Despite being less frequent than hypermethylation of CGI, hypomethylation of non‐CGI promoters can result in elevated expression of oncogenes and proto‐oncogenes [45]. Our data show that the CpG‐aggregated methylation analysis shows that USP22 is significantly hypomethylated in tumor samples compared to normal tissues across the majority of cancer types, which is highly associated with its known upregulation in cancer. The found hypomethylation of cg10779317 in tumors is consistent with the known overexpression of USP22 in many malignancies, where it functions as an oncogene, driving tumor development, treatment resistance, and poor prognosis. When USP22 is overexpressed, it is attracted to deubiquitinate H2A and H2B, which leads to higher gene activation and transcription and ultimately results in cancer development [18].

The analysis of the USP22 PPI network revealed that 16 nodes are directionally correlated with six proteins: C‐Myc, Lysine‐specific demethylase 1A (KDM1A), SIRT1, B‐lymphoma Mo‐MLV insertion region 1 (BMI1), cyclin B1 (CCNB1), and cyclin D1 (CCND1). Several independent studies revealed a significant connection between USP22 and these proteins in cancer. C‐Myc is generally associated with breast cancer, where patients showed hypermethylation of BRCA1, and together with C‐Myc overexpression, USP22 is said to be a positive regulator of C‐Myc [46]. USP22 deubiquitinates BMI‐1, inhibiting the expression of the cyclin‐dependent kinase inhibitor INK4A/B and promoting tumor growth. Additionally, BMI1 deubiquitination controls the gene expression linked to glioma stemness in both clinical tissue and glioma cell lines, including POST, HEY2, PDGFRA, and ATF3 [29, 47]. USP22 promotes cell cycle progression and the growth of cancer cells via modifying the stability of the CCNB1 protein. In colon cancer tissues, 7 out of 10 patients exhibited higher levels of both CCNB1 and USP22 protein expression compared to normal controls [48, 49]. The GSK3β‐USP22‐KDM1A axis plays a vital role in glioblastoma carcinogenesis. Following CK1α's phosphorylation of KDM1A serine 687, GSK3β phosphorylates serine 683. Phosphorylation of KDM1A results in interaction with USP22, and its deubiquitinating activity results in KDM1A stability [50, 51]. USP22 stabilizes SIRT1 by deubiquitinating it, which prevents p53 activity and transcriptional initiation of p53 target genes and suppresses cell death [52]. These early studies agree with our correlation study. Protein kinases and other enzymes that control protein phosphorylation have been shown to be an appropriate entry point for new anticancer treatments [53]. The ubiquitin system has been lagging behind in drug development efforts due to the complexity of the ubiquitin‐conjugating and deconjugating mechanisms and the lack of understanding of many aspects of the biology of this pathway, especially the topology of polyubiquitin chains and posttranslational modifications present on ubiquitin itself [54]. Our data support the observation of the previous studies [19, 33, 44, 49, 55, 56, 57] that USP22 may not be the sole initiator of tumorigenesis, but it exhibits an oncogenic role with other oncogenic factors. Its dual nature as an oncogene and in some contexts as a tumor suppressor reflects its complex functionality, which varies by cellular environment and tumor type, positioning USP22 as a challenging therapeutic target. These findings suggest that USP22 may not function as an independent driver of tumor progression; its oncogenic potential may be exerted through interaction with a key regulatory protein.

Nevertheless, different databases may use different techniques for gathering and processing data, which might result in systematic biases. Inaccurate results might have been caused by small sample sizes for several rare tumor forms. For example, UALCAN had insufficient quantities of normal material for some tumor types in proteomic, methylation, and expression studies.

## 5 Conclusion

In summary, the present study concluded that USP22 is expressed in 13 cancer types and shows significant correlation with poor survival in patient outcomes, and may be regarded as a generic tumor marker. Importantly, USP22 overexpression is associated with altered methylation patterns, suggesting an epigenetic mechanism that may drive its oncogenic role. USP22 plays a multifaceted role in cancer, contributing to tumor biology through its deubiquitination activity and stabilizing various proteins like BMI1, Cyclin B1, Cyclin D1, survivin, and SIRT1. However, its effects appear to be highly context‐dependent and mediated through its interaction with multiple oncogenic partners. This study solely employed a bioinformatics method, which yielded the first evidence connecting USP22 to the development of cancer in a variety of malignancies. Additional in vitro or in vivo experimental studies are further needed to strongly prove the pan‐cancer role of USP22 and its potential as a therapeutic target across diverse.

## Author Contributions

**Uma Devi A.:** conceptualization, investigation, writing – original draft, methodology, validation, visualization, writing – review and editing, formal analysis, data curation, resources, software. **Prakash Kumar Shukla:** conceptualization, investigation, writing – original draft, validation, methodology, visualization, writing – review and editing, formal analysis, project administration, supervision, data curation.

## Funding

The authors gratefully acknowledge Vellore Institute of Technology for providing financial support for this work. P.K.S. acknowledges the Department of Biotechnology, India for the BT/RLF/Re‐entry/28/2022 fellowship.

## Ethics Statement

The authors have nothing to report.

## Conflicts of Interest

The authors declare no conflicts of interest.

## Supporting information


**Figure S1:** Protein expression level of USP22 in different types of cancer. (A) USP22 protein level was upregulated in breast cancer, clear cell RCC, lung cancer, head and neck cancer, glioblastoma, and liver cancer, acquired using the UALCAN web server with the CPTAC dataset, and *Z* values are obtained by normalizing log_2_ spectral count ratios both within and between CPTAC samples (*p < 0.05, **p < 0.01, and ***p < 0.001). (B) Immunohistochemical staining results from the HPA dataset of different cancer types.
**Figure S2:** Expression of USP22 in different pathological cancer stages did not identify USP22 as a significantly differentially expressed gene. Statistical analysis was performed using one ANOVA analysis as implemented in the UALCAN platform; statistical significance was annotated by stars (*p < 0.05, **p < 0.01, and ***p < 0.001).
**Figure S3:** USP22 expression in histological subtypes. Statistical analysis was performed using one ANOVA analysis as implemented in the UALCAN platform; statistical significance was annotated by stars (*p < 0.05, **p < 0.01, and ***p < 0.001).
**Figure S4:** Expression of USP22 level in various cancer tumor grades. Statistical analysis was performed using one ANOVA analysis as implemented in the UALCAN platform; statistical significance was annotated by stars (*p < 0.05, **p < 0.01, and ***p < 0.001).
**Figure S5:** Expression level of USP22 in different cancer types in accordance with TP53 mutation status. Statistical analysis was performed using one ANOVA analysis as implemented in the UALCAN platform; statistical significance was annotated by stars (*p < 0.05, **p < 0.01, and ***p < 0.001).
**Figure S6:** Correlation between USP22 expression and survival probability of patients with different tumors. Kaplan–Meier survival analysis was utilized to determine the impact of gene expression on patient survival. Patients were classified based on expression levels, with high expression corresponding to values more than the third quartile. Statistical significance was determined using the log‐rank test.
**Figure S7:** Survival plot of samples with alterations/mutations in USP22. (A) Overall survival of USP22. (B) Disease‐specific survival of USP22, as utilized using the cBioPortal tool. The Kaplan–Meier technique is used in this survival plot to compare patients with and without mutation (overall survival: *n* = 228 vs. 10 575, Disease‐specific: *n* = 222 vs. 10 036). A log‐rank test was used to assess the significance, with hazard ratios calculated at a 95% confidence level.
**Figure S8:** Forest plot of multivariate Cox regression of KIRC.
**Figure S9:** Forest plot of multivariate Cox regression of KIRP.
**Figure S10:** Forest plot of multivariate Cox regression of LIHC.
**Figure S11:** Forest plot of multivariate Cox regression of PAAD.

**Table S1:** Association of USP22 expression with individual stages, tumor grade, histological subtypes, and TP53 mutation stage using the UALCAN web server.
**Table S2:** This table highlights protein alterations, mutation types, copy number variations, and allele frequencies in TCGA cancer samples having USP22‐related mutation events across several cancer types.
**Table S3:** Hazard ratio of the overall survival rate.
**Table S4:** Survival plot summary.
**Table S5:** Hazard ratio for disease‐specific survival.
**Table S6:** Summary of disease‐specific plot.

## Data Availability

All data generated and analyzed during this study are publicly available in relevant online repositories. The names of those repositories, along with the accession number, are provided in the article. The information that underpins the conclusions of this research can be obtained from the corresponding author upon request.
