# Supplementary material for: Decoding the Oncogenic Role of USP22 Through Pan‐Cancer Genomic and Epigenetic Analysis
Source: Cancer Rep (Hoboken). 2026 May 13;9(5):e70572. doi: 10.1002/cnr2.70572 (PMC13171463; doi:10.1002/cnr2.70572)

**Supplementary file**

**Figure S1:** Protein expression level of USP22 in different types of cancer. A) USP22 protein level was upregulated in breast cancer, clear cell RCC, lung cancer, head and neck cancer, Glioblastoma, and Liver cancer, acquired using the UALCAN webserver with the CPTAC dataset and Z values are obtained by normalizing log2 spectral count ratios both within and between CPTAC samples (*: p-value < 0.05; **: p-value <0.01; ***: p-value <0.001). B) Immunohistochemical staining results from the HPA dataset of different cancer types.


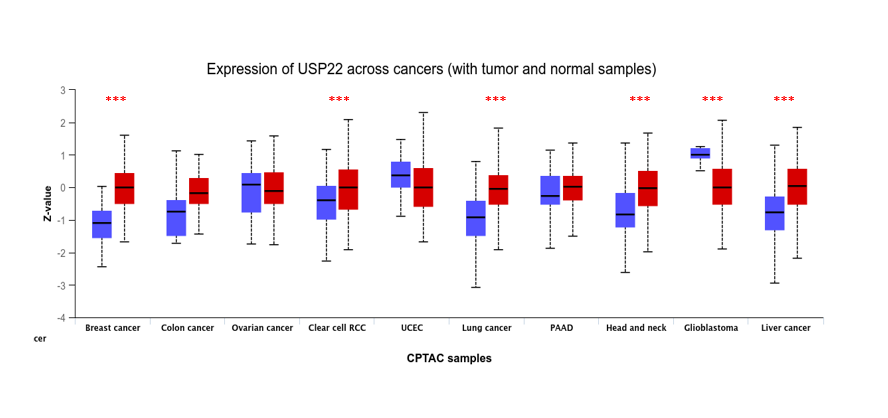


**
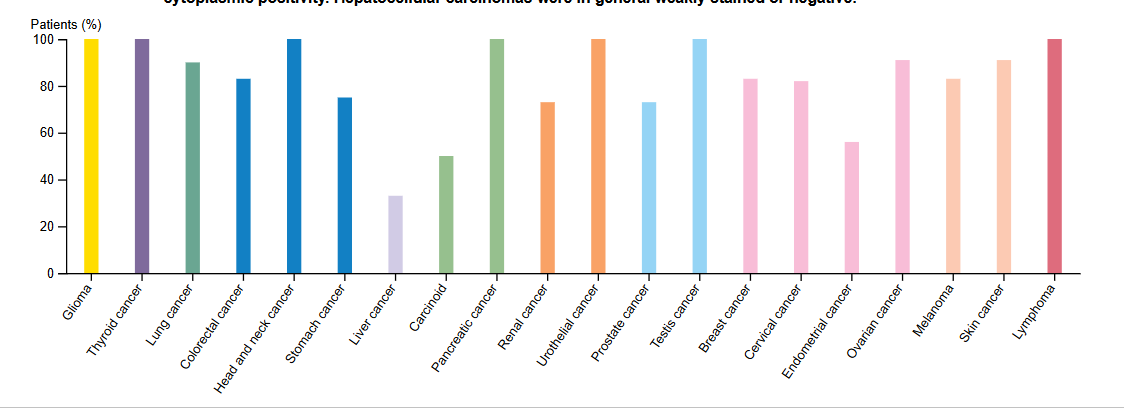
**

**Figure S2**: Expression of USP22 in different pathological cancer stages, that didn’t identify USP22 as a significantly differentially expressed gene. Statistical analysis was performed using one ANOVA analysis as implemented in the UALCAN platform; statistical significance was annotated by stars (*: p-value < 0.05; **: p-value <0.01; ***: p-value <0.001).


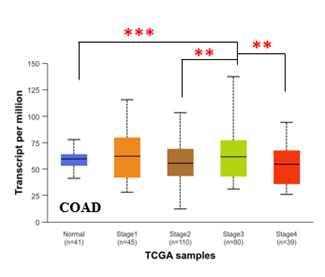

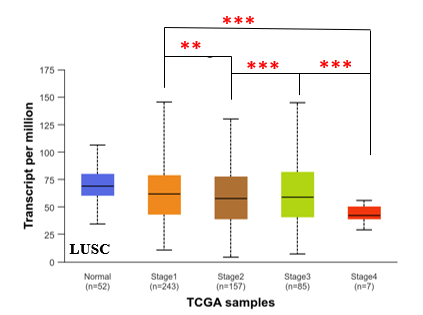

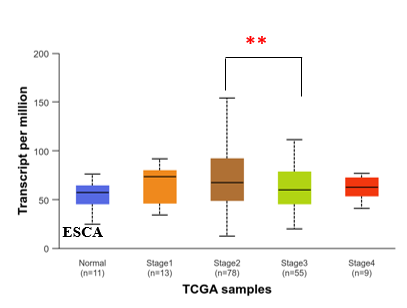

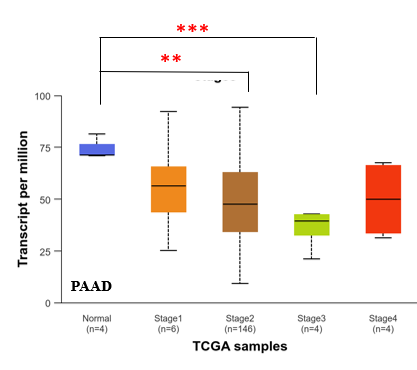

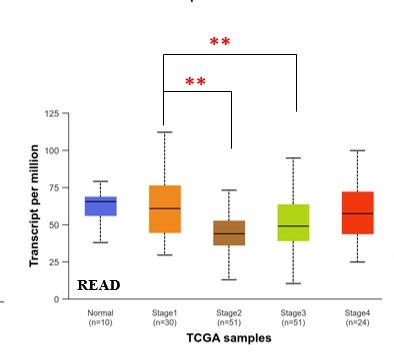

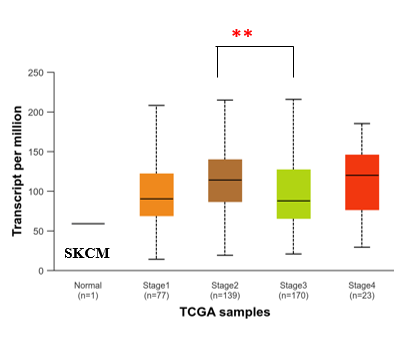


**Figure S3**: USP22 expression in Histological subtypes. Statistical analysis was performed using one ANOVA analysis as implemented in the UALCAN platform; statistical significance was annotated by stars (*: p-value < 0.05; **: p-value <0.01; ***: p-value <0.001).


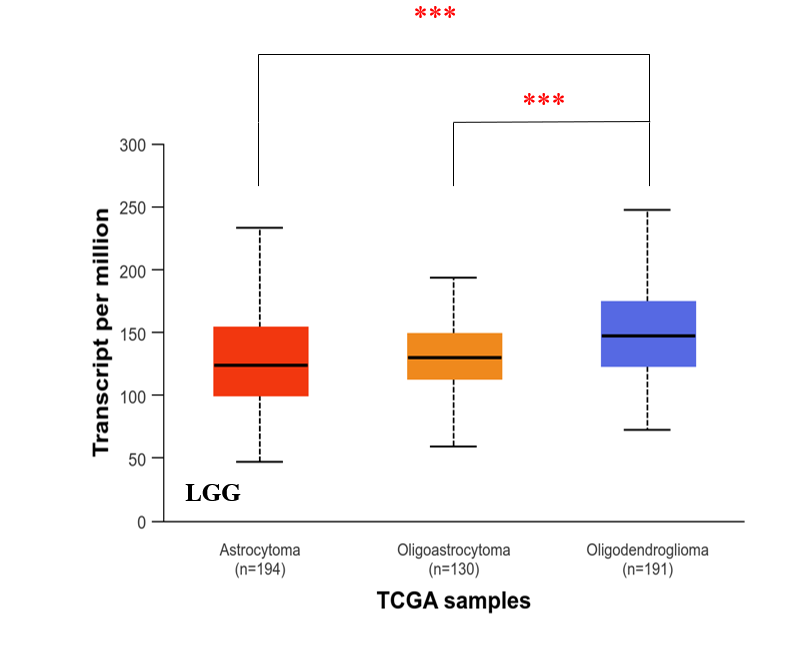

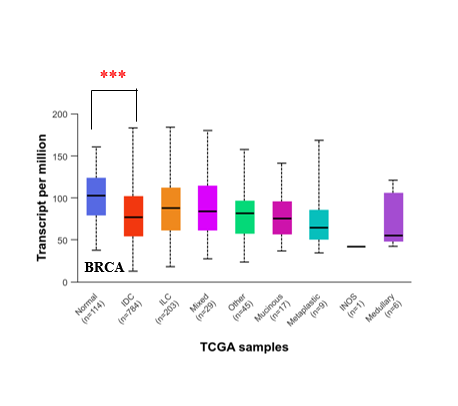

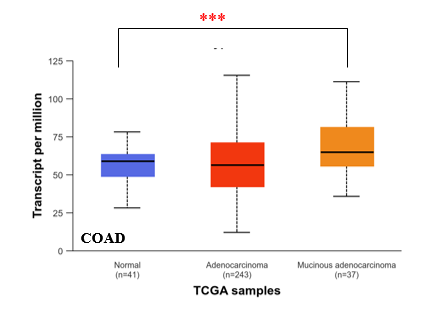

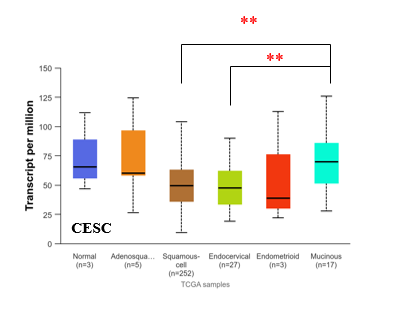

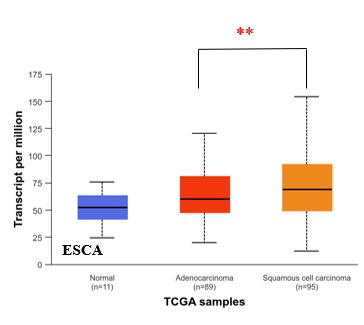

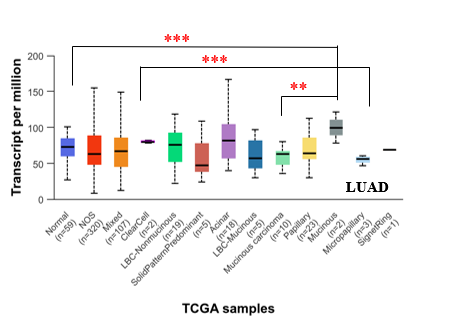

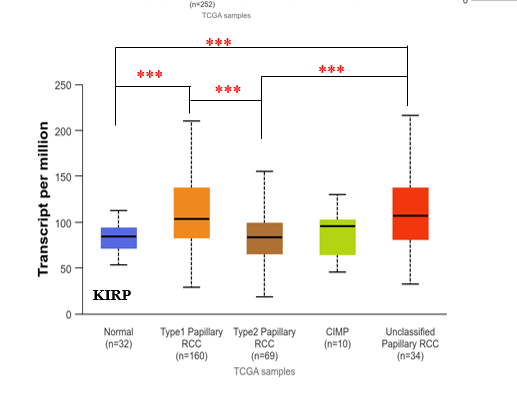

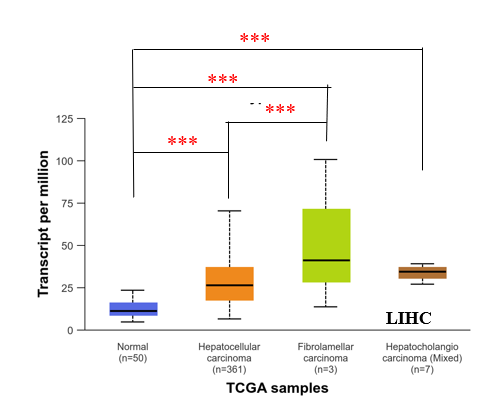

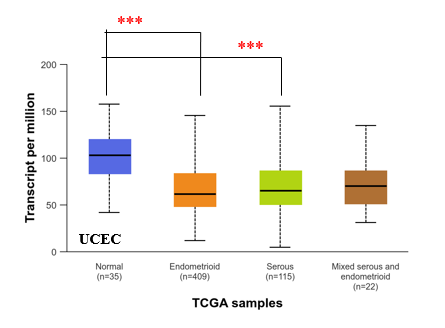

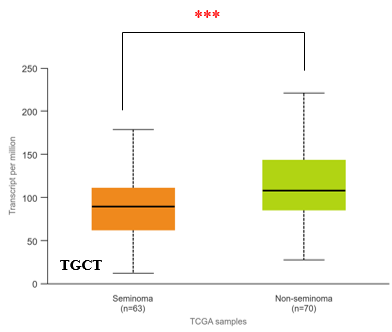

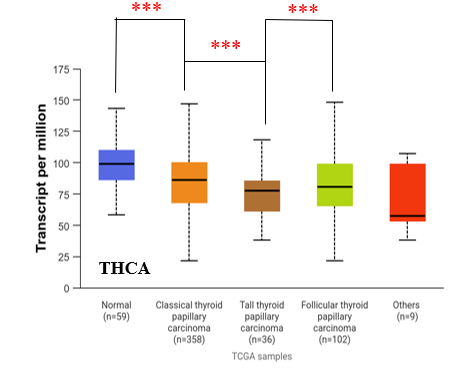

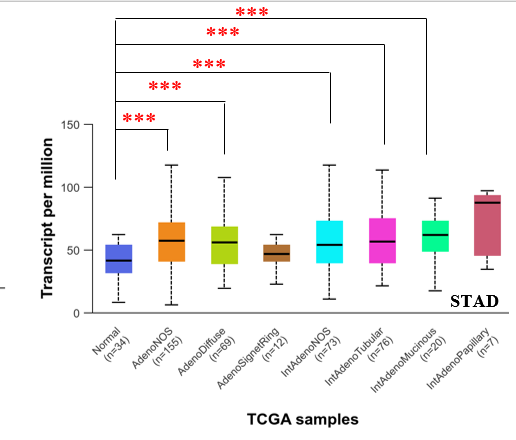


**Figure S4**: Expression of USP22 level in various cancer tumor grades. Statistical analysis was performed using one ANOVA analysis as implemented in the UALCAN platform; statistical significance was annotated by stars (*: p-value < 0.05; **: p-value <0.01; ***: p-value <0.001).


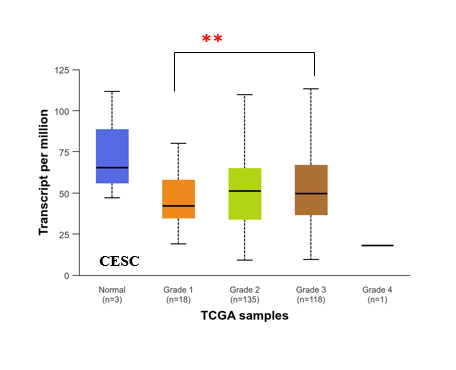

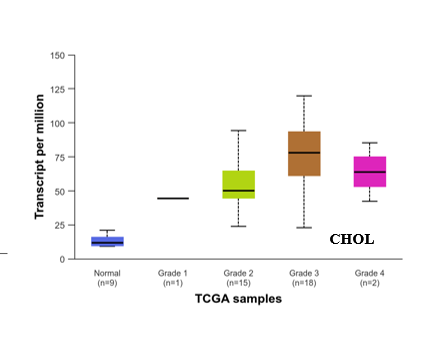

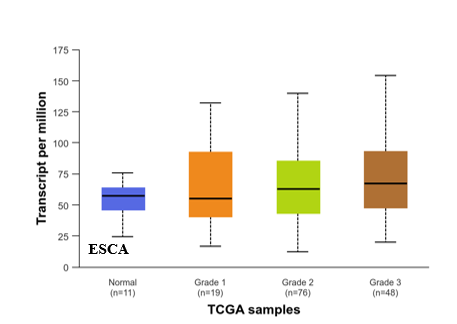


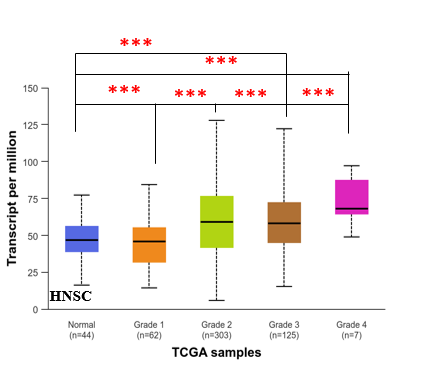

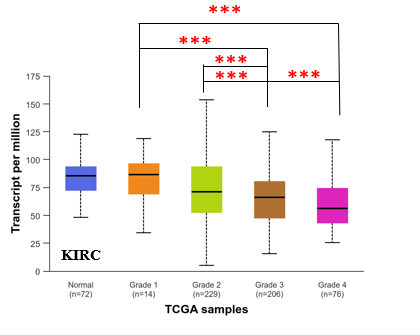

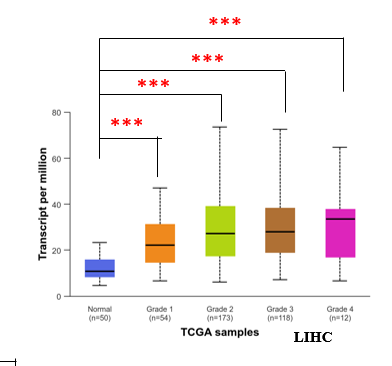


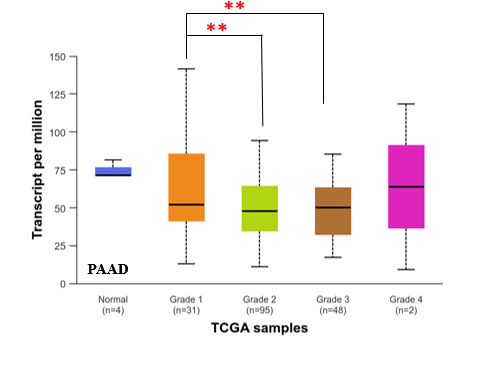

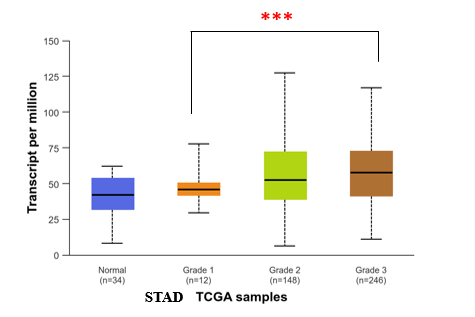


**Figure S5**: Expression level of USP22 in different cancer types in accordance to TP53 mutation status. Statistical analysis was performed using one ANOVA analysis as implemented in the UALCAN platform; statistical significance was annotated by stars (*: p-value < 0.05; **: p-value <0.01; ***: p-value <0.001).


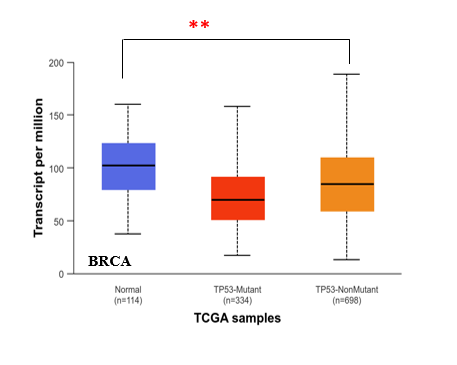

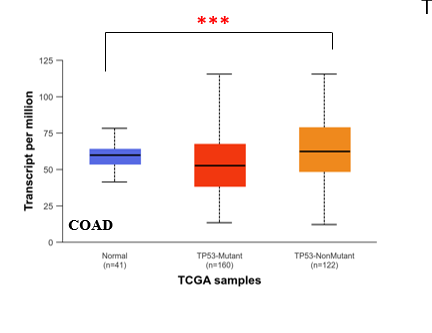

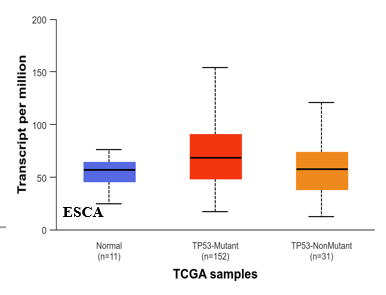


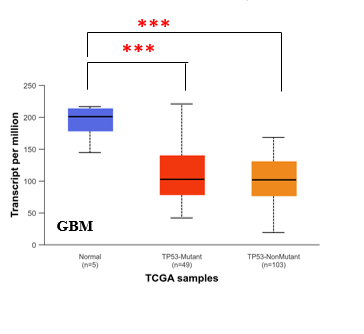

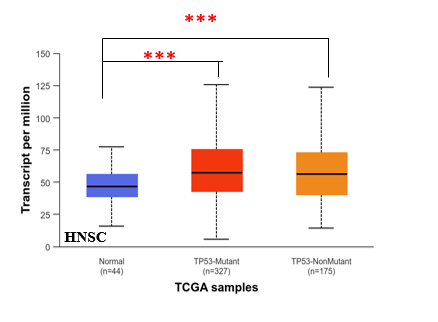

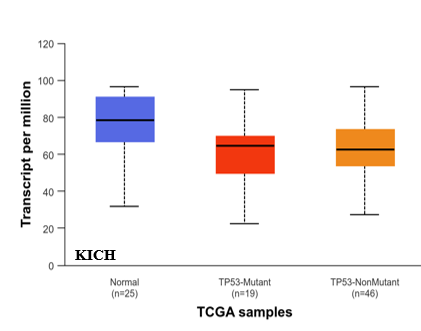


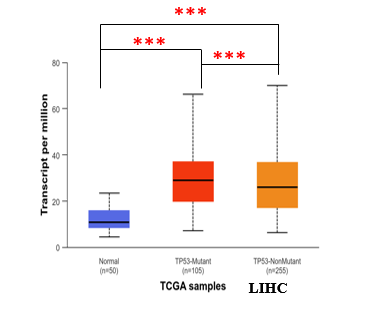

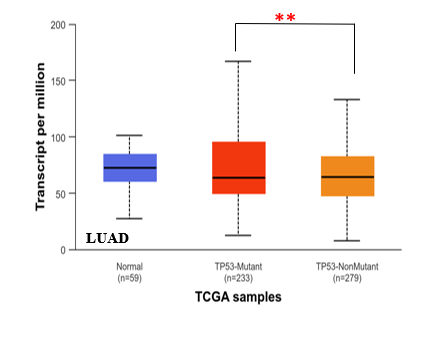

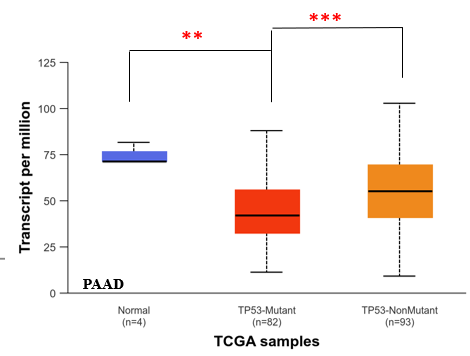


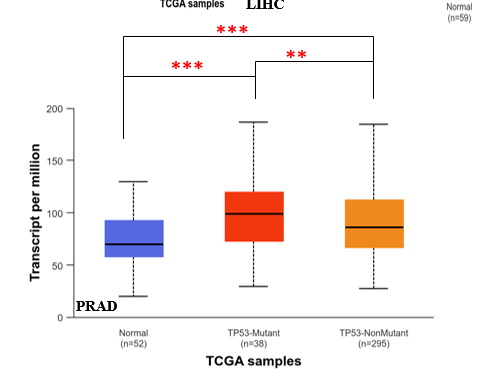

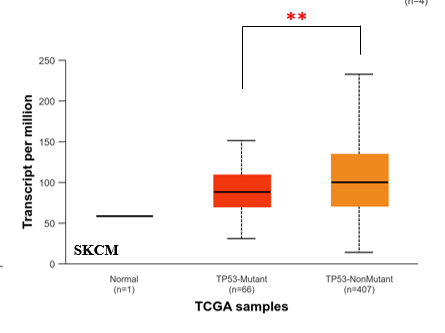

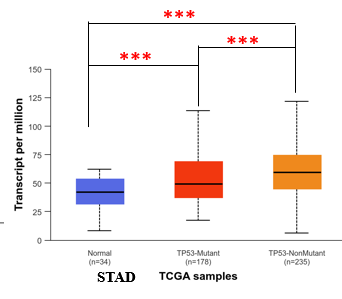


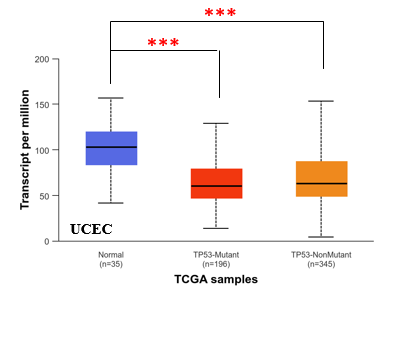


**Figure S6**: Correlation between USP22 expression and survival probability of patients with different tumors. Kaplan-Merier survival analysis was utilized to determine the impact of gene expression on patient survival. Patients were classified based on expression levels, with high expression corresponding to values more than the third quartile. Statistical significance was determined using the log-rank test.


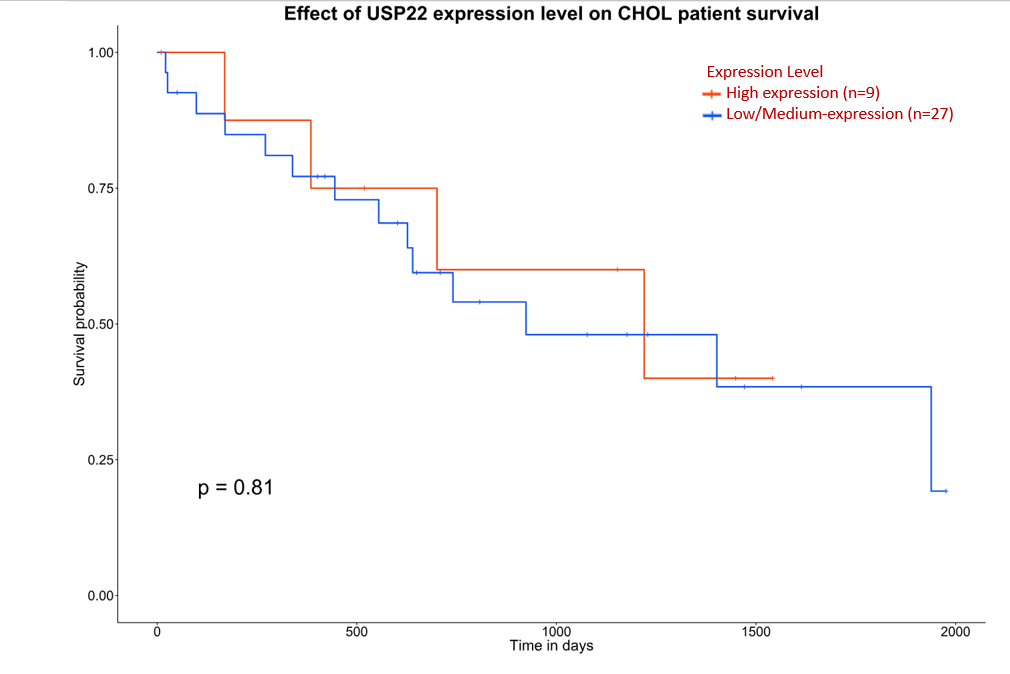

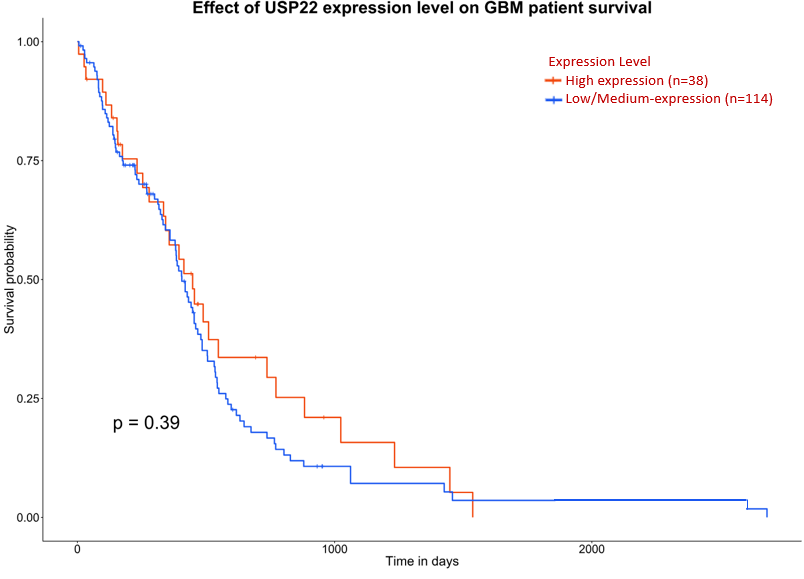


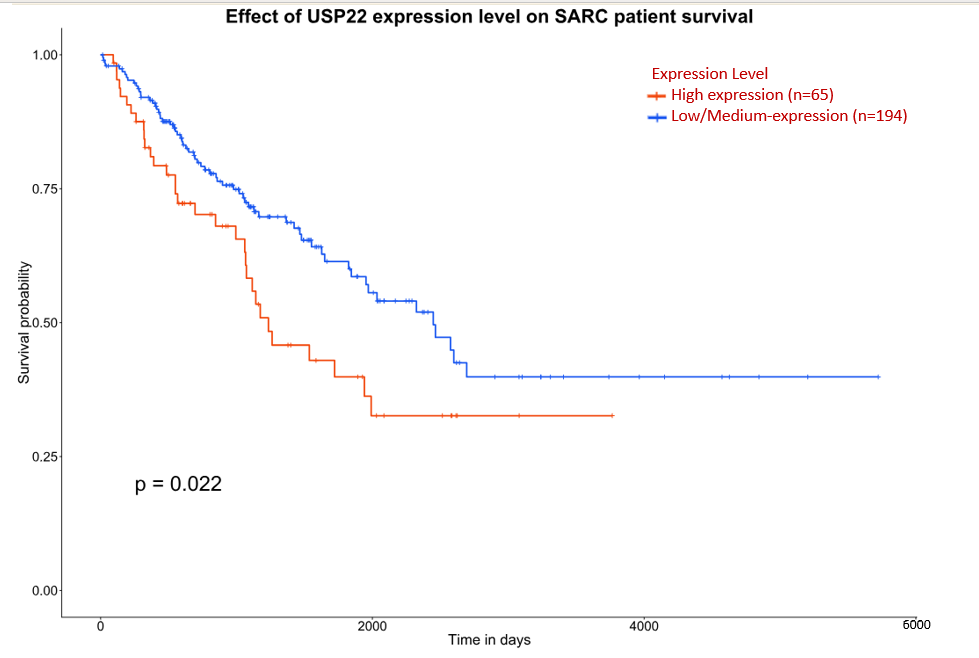

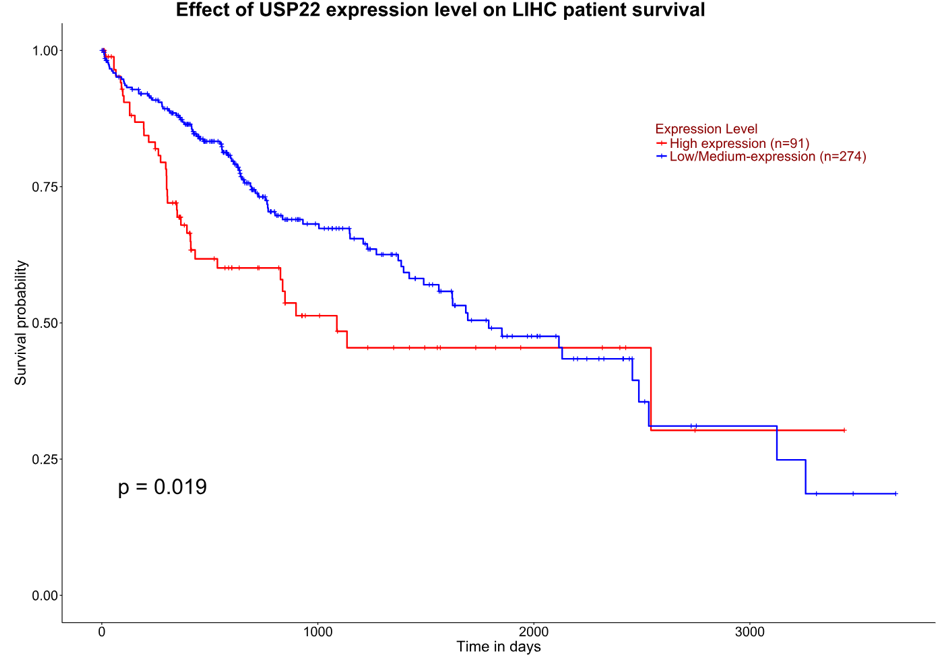


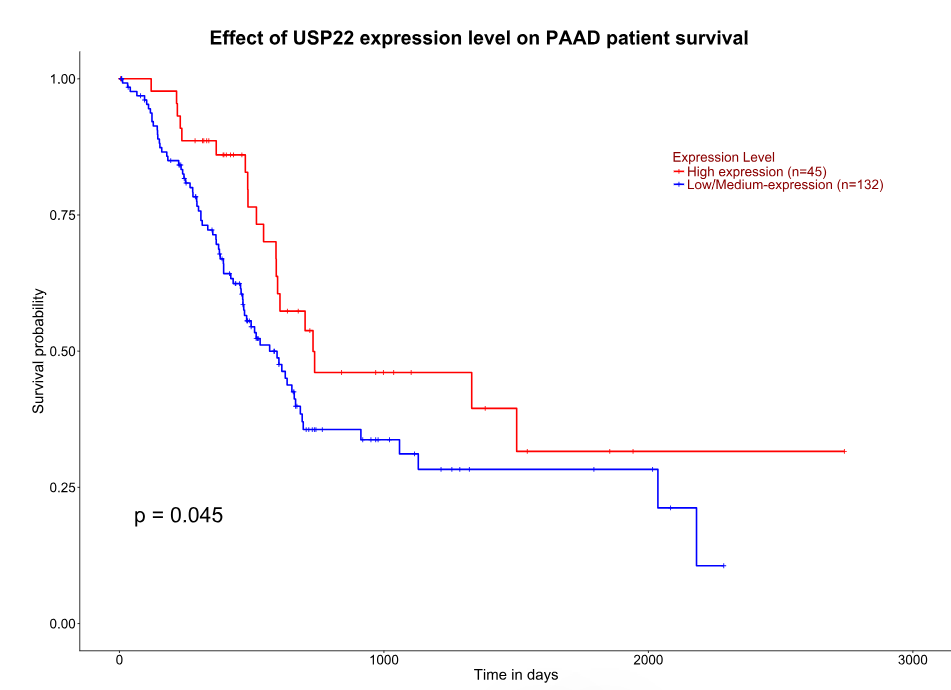

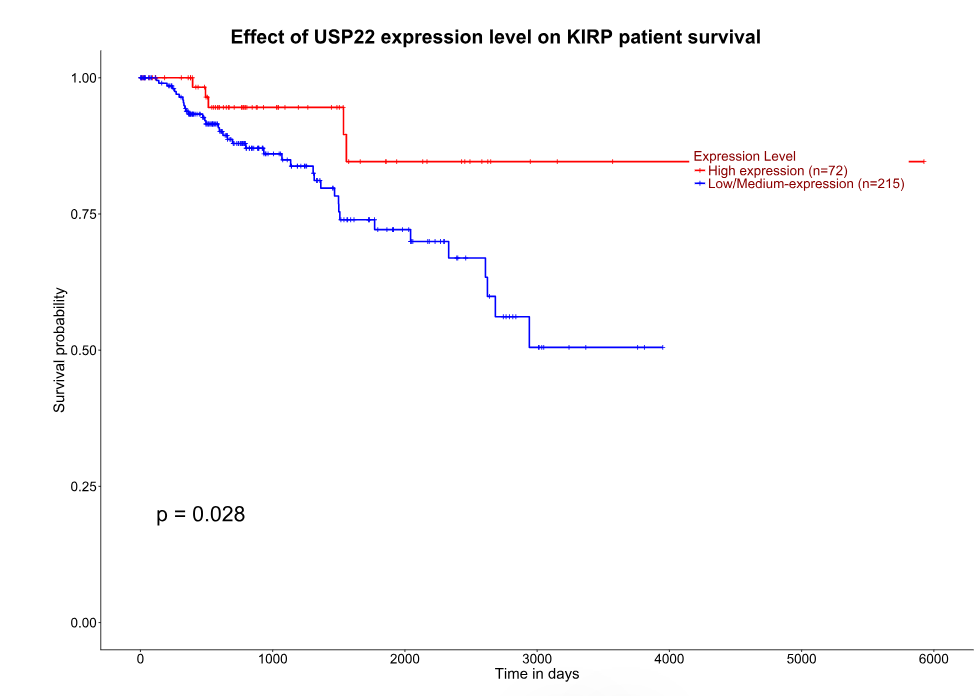


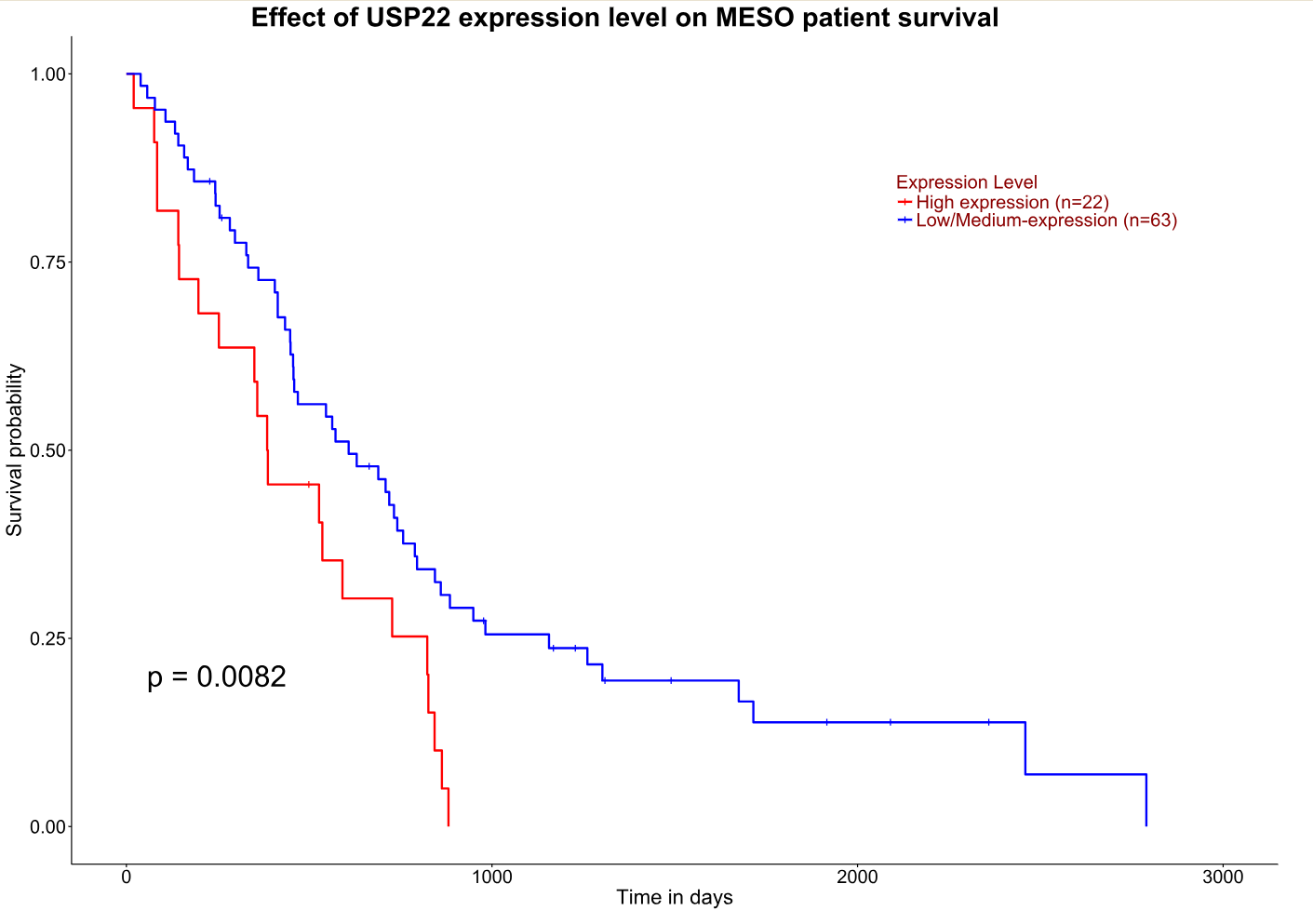

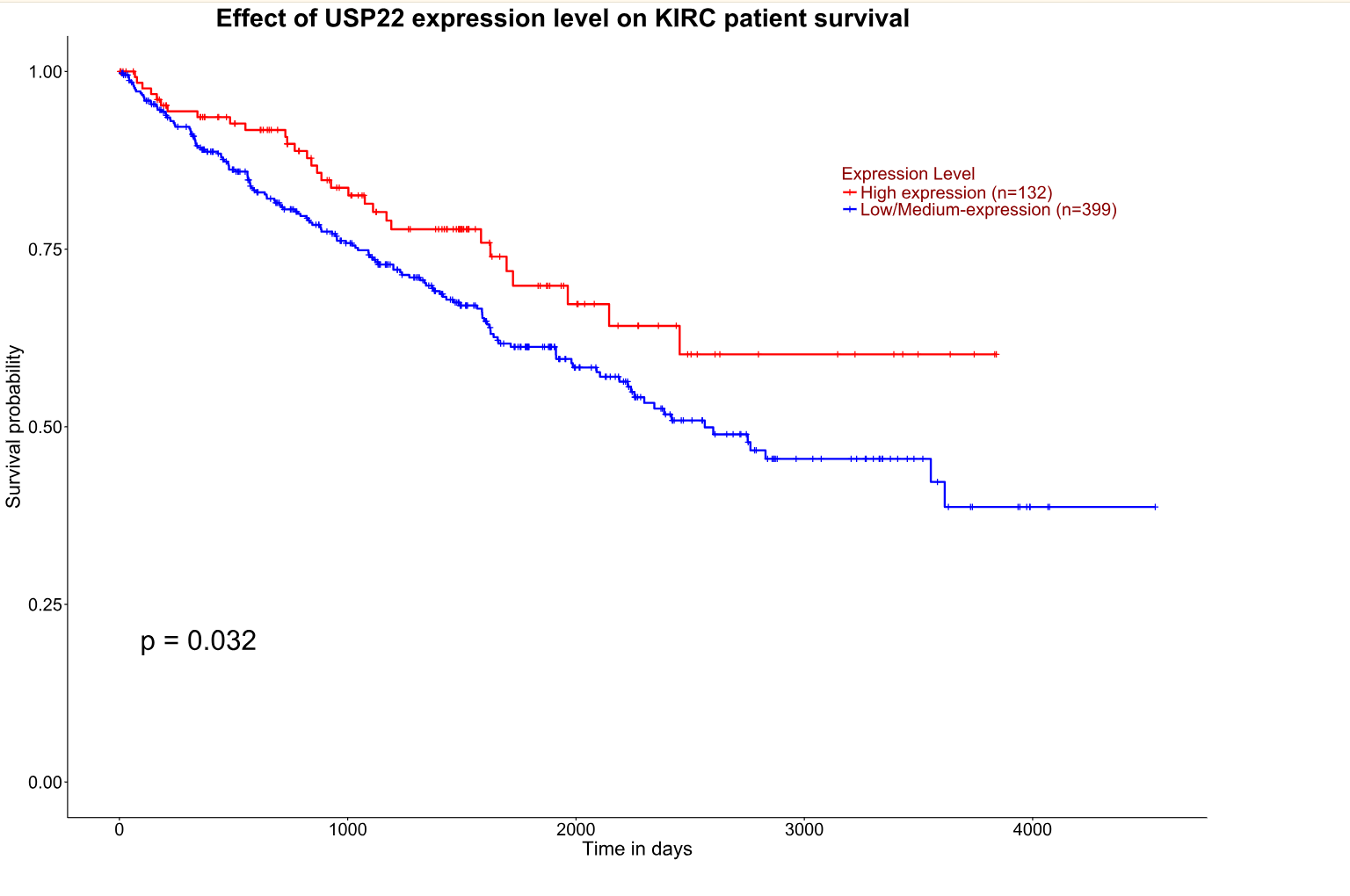


**Figure S7**: Survival plot of samples with alterations/mutations in USP22. A) Overall survival of USP22, B) Disease-specific survival of USP22, as utilized using the cBioportal tool. The Kaplan-Meier technique is used in this survival plot to compare patients with and without mutation (Overall survival: n=228 vs 10,575, Disease-specific: n=222 vs 10036). A log-rank test was used to assess the significance, with hazard ratios calculated at a 95% confidence level.

A)


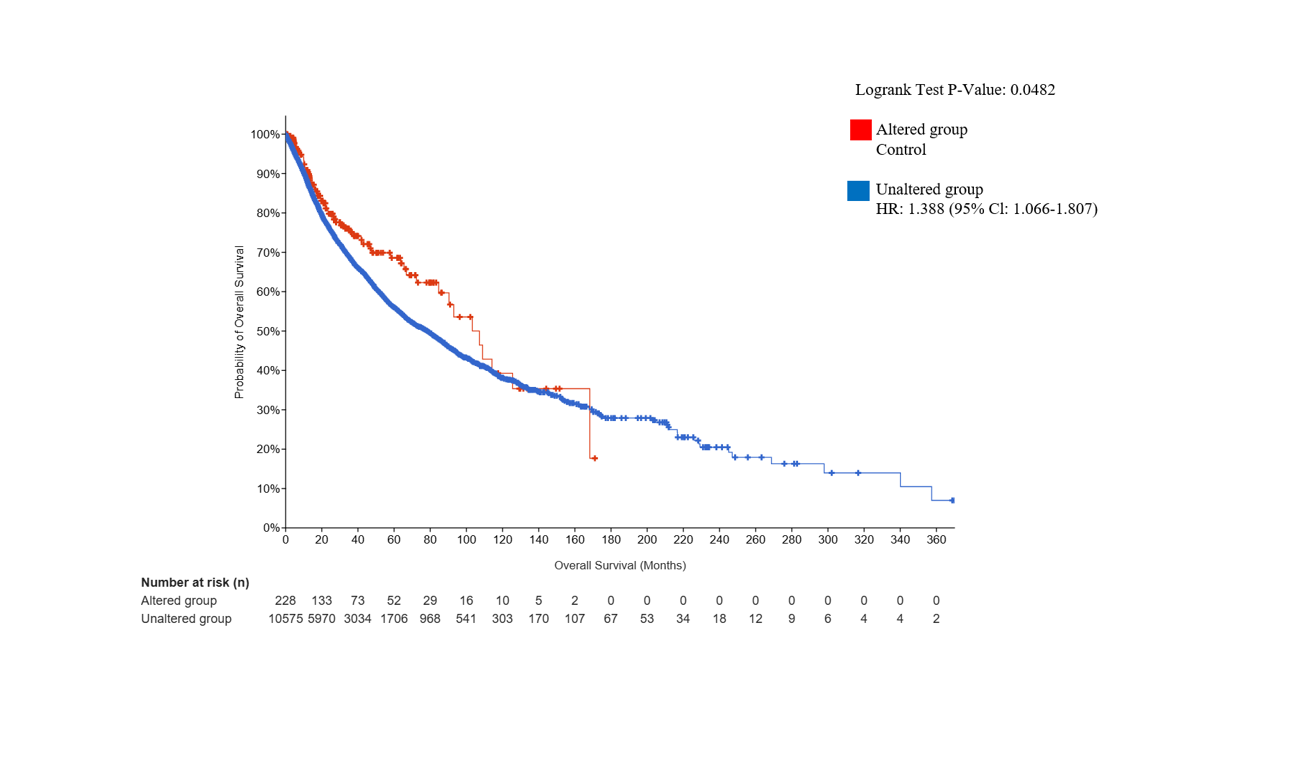


B)


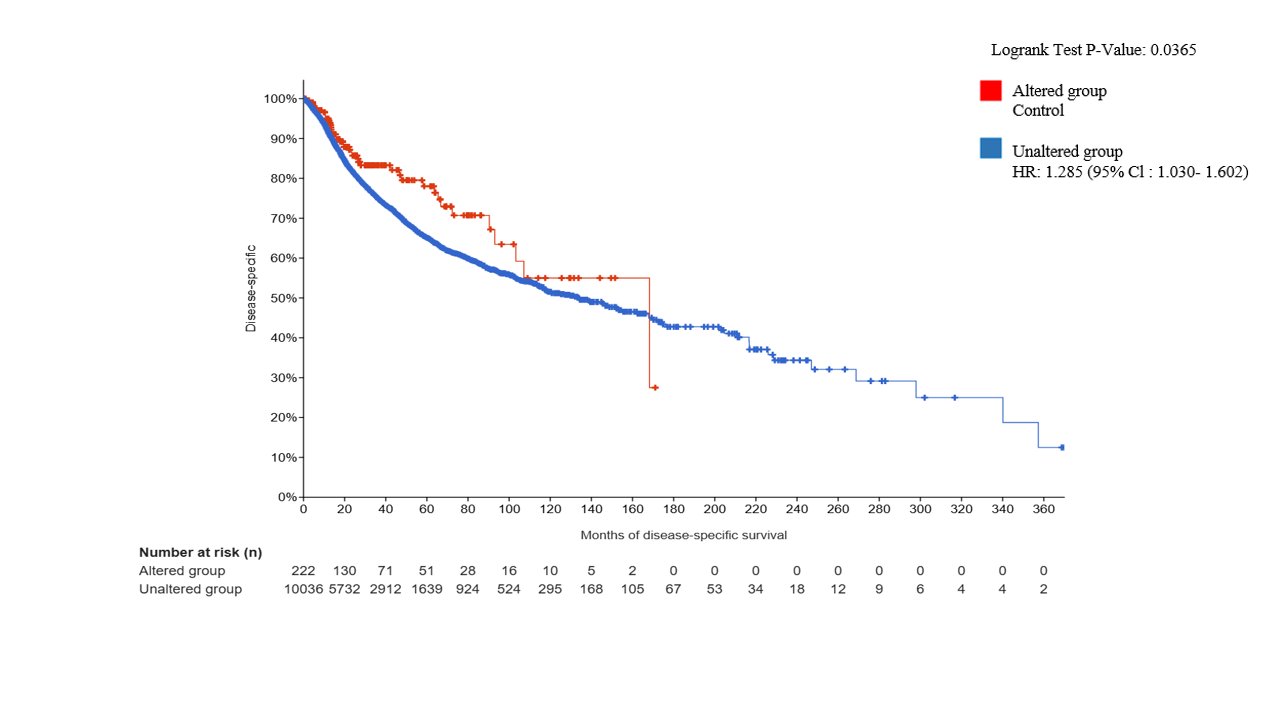


Figure S8: Forest plot of multivariate Cox regression of KIRC


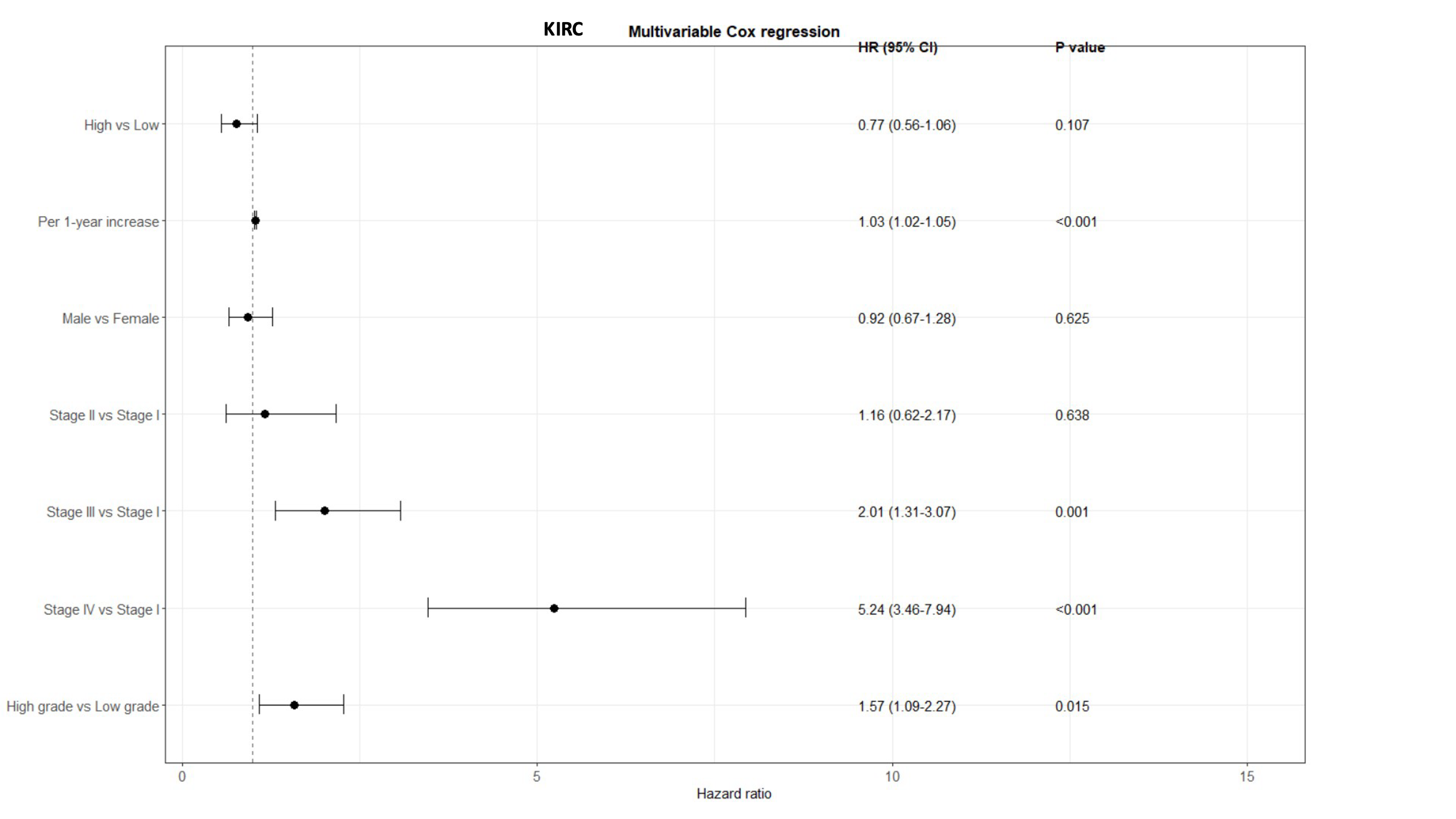


Figure S9: Forest plot of multivariate Cox regression of KIRP


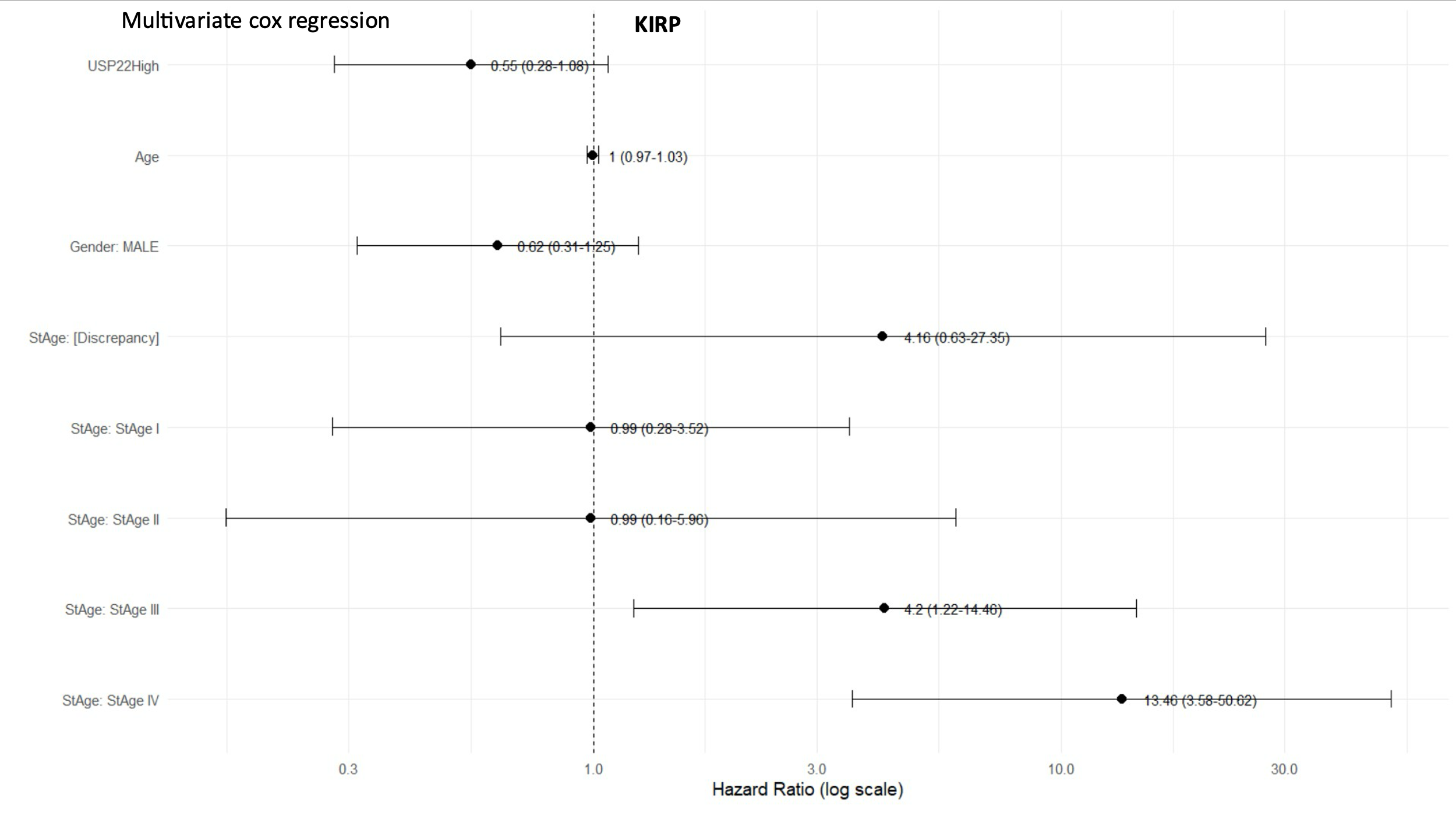


Figure S10: Forest plot of multivariate Cox regression of LIHC


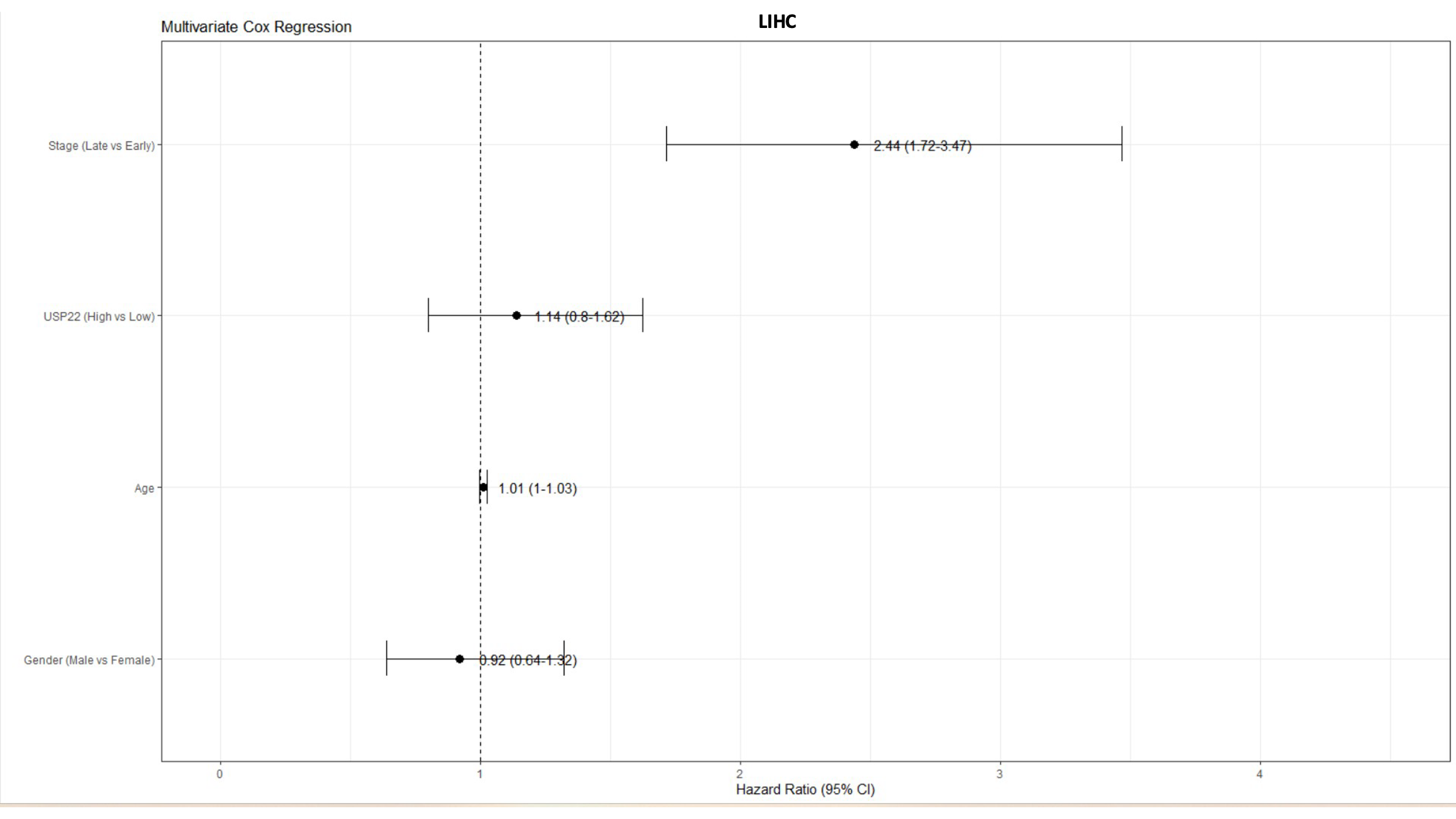


Figure S11: Forest plot of multivariate Cox regression of PAAD

Multivariate Cox regression


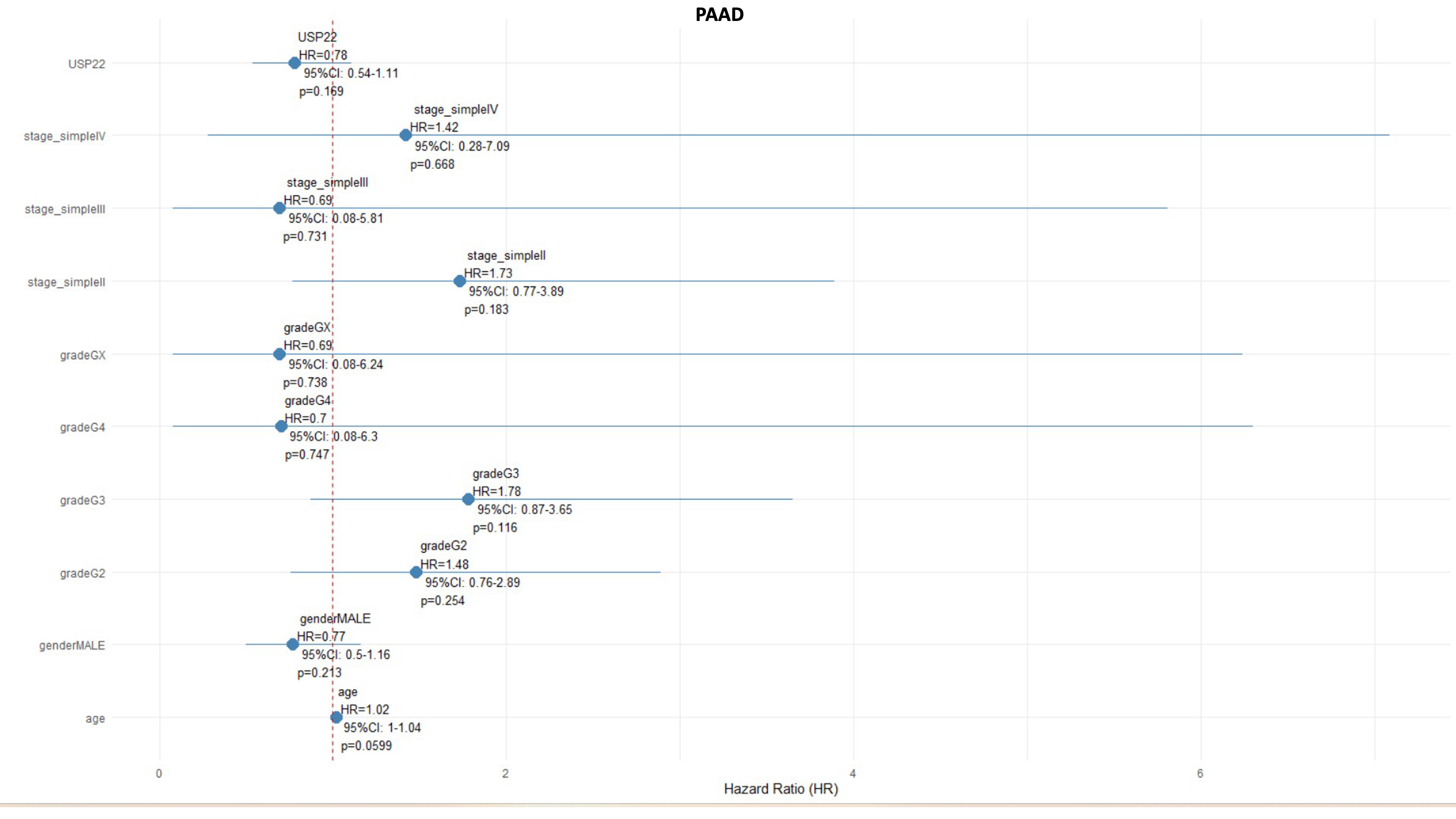

Supplement: Supplementary file 1 — Figure S1: Protein expression level of USP22 in different types of cancer. (A) USP22 protein level was upregulated in breast cancer, clear cell RCC, lung cancer, head and neck cancer, glioblastoma, and liver cancer, acquired using the UALCAN web server with the CPTAC dataset, and Z values are obtained by normalizing log2 spectral count ratios both within and between CPTAC samples (*p < 0.05, **p < 0.01, and ***p < 0.001). (B) Immunohistochemical staining results from the HPA dataset of different cancer types. Figure S2: Expression of USP22 in different pathological cancer stages did not identify USP22 as a significantly differentially expressed gene. Statistical analysis was performed using one ANOVA analysis as implemented in the UALCAN platform; statistical significance was annotated by stars (*p < 0.05, **p < 0.01, and ***p < 0.001). Figure S3: USP22 expression in histological subtypes. Statistical analysis was performed using one ANOVA analysis as implemented in the UALCAN platform; statistical significance was annotated by stars (*p < 0.05, **p < 0.01, and ***p < 0.001). Figure S4: Expression of USP22 level in various cancer tumor grades. Statistical analysis was performed using one ANOVA analysis as implemented in the UALCAN platform; statistical significance was annotated by stars (*p < 0.05, **p < 0.01, and ***p < 0.001). Figure S5: Expression level of USP22 in different cancer types in accordance with TP53 mutation status. Statistical analysis was performed using one ANOVA analysis as implemented in the UALCAN platform; statistical significance was annotated by stars (*p < 0.05, **p < 0.01, and ***p < 0.001). Figure S6: Correlation between USP22 expression and survival probability of patients with different tumors. Kaplan–Meier survival analysis was utilized to determine the impact of gene expression on patient survival. Patients were classified based on expression levels, with high expression corresponding to values more than the third quartile. Statistica [file CNR2-9-e70572-s001.docx]
