# Supplementary material for: Decoding the Oncogenic Role of USP22 Through Pan‐Cancer Genomic and Epigenetic Analysis
Source: Cancer Rep (Hoboken). 2026 May 13;9(5):e70572. doi: 10.1002/cnr2.70572 (PMC13171463; doi:10.1002/cnr2.70572)
Supplement: Supplementary file 2 — Table S1: Association of USP22 expression with individual stages, tumor grade, histological subtypes, and TP53 mutation stage using the UALCAN web server. Table S2: This table highlights protein alterations, mutation types, copy number variations, and allele frequencies in TCGA cancer samples having USP22‐related mutation events across several cancer types. Table S3: Hazard ratio of the overall survival rate. Table S4: Survival plot summary. Table S5: Hazard ratio for disease‐specific survival. Table S6: Summary of disease‐specific plot. [file CNR2-9-e70572-s002.docx]

Supplementary tables

Table S1: Association of USP22 expression with individual stages, tumor grade, histological subtypes, and TP53 mutation stage using the UALCAN web server

| Tumor | Individual cancer stages | Tumor grade | Histological subtypes | TP53 mutation stage |
| --- | --- | --- | --- | --- |
| LGG |  |  | Astrocytoma vs Oligoastrocytoma p=0.2  Astrocytoma vs Oligodendroglioma p=3 ×10^-^7  Oligoastrocytoma vs Oligodendroglioma p=0.00033 |  |
| BRCA | N vs S1 P=0.038  N vs S2 P=0.25  N vs S3 P=0.0030  N vs S4 P=0.7  S1 vs S2 P= 0.62  S1 vs S3 P=0.4  S1 vs S4 P=0.7  S2 vs S3 P=0.2  S2 vs S4 P=0.93  S3 vs S4 P= 0.53 |  | N vs Infiltrating Ductal Carcinoma P=0.0025 | N vs TP53 mutant P=0.29  N vs TP53-Non mutant P=0.001  TP53-mutant vs TP53-Nonmutant P=0.923 |
| CESC |  | Grade 1 vs Grade 2 P= 0.155  Grade 1 vs Grade 3 P= 0.02  Grade 2 vs Grade 3 P=0.33 | Squamous cell VS Mucinous P=0.0029  Endocervical vs Mucinous P=0.002 |  |
| CHOL | N vs S1 P=1×10^-^7  N vs S2 P= 0.00031  N vs S4 P=0.00022  S1 vs S2 P=0.6  S1 vs S4 P=0.51  S2 vs S4 P=0.2 | N vs Grade 2 P= 0.9  N vs Grade 3 P=0.6  N vs Grade 4 P= 1  Grade 2 vs Grade 3 p=0.19  Grade 2 vs Grade 4 P=0.90  Grade 3 vs Grade 4 P=0.62 |  |  |
| COAD | N vs S1 P=0.16  N vs S2 P=0.48  N vs Stage 3 P=0.0075  N vs S4 P=0.93  S1 VS S2 P=0.41  S1 vs S3 P=0.16  S1 vs S4 P=0.25  S2 vs S3 P=0.028  S2 vs S4 P=0.59  S3 vs S4 P=0.021 |  | N vs Adenocarcioma P= 0.061  N vs Mucinous adenocarcinoma P=0.0026  Adenocarcinoma vs Mucinous adenocarcinoma P=0.097 | N vs TP53 mutant P=0.24  N vs TP53 Non mutant P=0.0089  TP53 mutant vs TP53 Non mutant P=0.36 |
| ESCA | N vs S1 P=0.40  N vs S2 P= 0.28  N vs S3 p=0.80  N vs S4 P=0.25  S1 vs S2 P=0.86  S1 vs S3 P=0.267  S1 vs S4 P=0.70  S2 vs S3 P=0.04  S2 VS S4 P=0.77  S3 vs S4 P=0.12 | N vs G1 P=0.62  N vs Grade 2 P=0.43  N vs Grade 3 P=0.29  Grade 1 vs Grade 2 P=0.80  Grade 1 vs Grade 3 P=0.49  Grade 2 vs Grade 3 P=0.46 | N vs Adenocarcinoma P=0.31  N vs Squamous cell carcinoma P= 0.11  Adenocarcinoma vs Squamous cell carcinoma P=0.035 | N vs TP53 mutant P=0.26  N vs TP53 non mutant P=0.86  TP53 mutant vs TP53 Non mutant P=0.11 |
| GBM |  |  |  | N vs TP53 mutant P=0.0020  N vs TP53 Non mutant P=3×10^-^5  TP53 Mutant Vs TP53 Non mutant P=0.21 |
| HNSC | N vs S1 P=0.0019  N vs S2 P=0.0001  N vs S3 P=0.00090  N vs S4 P=6×10^-7^  S1 vs S2 P=0.9  S1 vs S3 P=0.73  S1 vs S4 P=0.87  S2 vs S3 P=0.72  S2 vs S4 P=0.65  S3 vs S4 P=0.43 | N vs Grade 1 P=0.56  N vs Grade 2 P=2×10^-8^  N vs Grade3 P= 6×10^-8^  N vs Grade 4 P=5 ×10^-5^  Grade 1 vs Grade 2 P=7.0×10^-7^  Grade 1 vs Grade 3 P=1×10^-6^  Grade 1 vs Grade 4 P=0.0011  Grade 2 vs Grade 3 P=0.46  Grade 2 vs Grade 4 P=0.29  Grade 3 vs Grade 4 P=0.40 |  | N vs TP53 mutant P= 4.3×10^-8^  N vs TP53 Non mutant P=1.5×10^-6^  TP53 mutant vs TP53 Non mutant P=0.62 |
| KICH | N vs S1 P=0.29  N vs S2 P=0.04  N vs S3 P=0.025  N vs S4 P=0.99  S1 vs S2 P=0.83  S1 vs S3 P=0.48  S1 vs S4 P=0.57  S2 vs S3 P=0.38  S2 vs S4 P=0.52  S3 vs S4 P=0.38 |  |  | N vs TP53mutant P=0.22  N vs TP53 Non mutant P=0.58  TP53 mutant vs TP53 non mutant P=0.74 |
| KIRC | N vs S1 P=0.0078  N vs S2 P=5 ×10^-7^  N vs S3 P=4 ×10^-11^  N vs S4 P=5×10^-7^  S1 vs S2 P=0.001  S1 vs S3 P=1×10^-6^  S1 vs S4 P=0.0004  S2 vs S3 P=0.86  S2 vs S4 P=0.954  S3 vs S4 P=0.92 | N vs Grade 1 P=0.555  N vs Grade 2 P=0.0013  N vs Grade 3 P=1×10^-9^  N vs Grade 4 P=2 ×10^-9^  Grade 1 VS Grade 2 P=0.07  Grade 1 vs Grade 3 P=0.002  Grade 1 vs Grade 4 P=0.0006  Grade 2 vs Grade 3 P=0.003  Grade 2 vs Grade 4 P=0.0003  Grade 3 vs Grade 4 P=0.10 |  |  |
| KIRP | N vs S1 P=3×10-^6^  N vs S2 P=0.06  N vs S3 P=0.07  N vs S4 P=0.1  S1 vs S2 P=0.5  S1 vs S3 P=0.57  S1 vs S4 P=0.01  S2 vs S3 P=0.92  S2 vs S4 P=0.02  S3 vs S4 P=0.053 |  | N vs Type 1 PRCC P<10^-9^  N vs Type 2 PRCC P=0.1  N vs KIRP CIMP P=0.7  N vs Unclassified PRCC P=0.00068  Type 1 PRCC vs Type 2 PRCC P=0.001  Type 1 PRCC vs KIRP CIMP P=0.07  Type 1 PRCC vs Unclassified PRCC P=0.4  Type 2 PRCC vs KIRP CIMP P=0.7  Type 2 PRCC vs unclassified PRCC P=0.005  KIRP CIMP vs Unclassified PRCC P=0.07 |  |
| LIHC | N vs S1 P<10^-12^  N vs S2 P<10^-12^  N vs S3 P<10^-12^  N vs S4 P<10^-7^  S1 vs S2 P=0.08  S1 vs S3 P=0.0001  S1 vs S4 P=0.8  S2 vs S3 P=0.03  S2 vs S4 P=0.13  S3 vs S4 P=0.006 | N vs Grade 1 P=2 ×10^-6^  N vs Grade 2 P<10^-12^  N vs Grade 3 P<10^-12^  N vs Grade 4 P=0.007  Grade 1 vs Grade 2 P=0.29  Grade 1 vs Grade 3 P=0.24  Grade 1 vs Grade 4 P=0.4  Grade 2 vs Grade 3 P=0.8  Grade 2 vs Grade 4 P=0.6  Grade 3 vs Grade 4 P=0.7 | N vs Hepatocellular carcinoma P<10^-12^  N vs Fibrolamellar carcinoma P=0.02  N vs Hepatocholangiocarcinoma (Mixed) P=1×10^-8^  Hepatocellular carcinoma vs Fibrolamellar carcinoma P=0.02  Hepatocellular carcinoma vs Hepatocholangio carcinoma (mixed) P=0.9  Fibrlamellar carcinoma vs Hepatocholangio carcinoma (Mixed) P=0.3 | N vs TP53 mutant P<10^-12^  N vs TP53 Non mutant P<10^-12^  TP53 mutant vs TP53 Non mutant P=0.04 |
| LUAD |  |  | N vs Mucinous P=0.03  Clear cell vs Micropapillary P=0.01  Mucinous carcinoma vs Mucinous P=0.03 | N vs TP53 mutant P=0.1  N vs TP53 Non mutant P=0.6  TP53 mutant vs TP53 Non mutant P=0.03 |
| LUSC | N vs S1 P=0.7  N vs S2 P=0.09  N vs S3 P=0.7  N vs S4 P=0.001  S1 vs S2 P=0.04  S1 vs S3 P=0.5  S1 vs S4 P=2 ×10^-5^  S2 vs S3 P=0.36  S2 vs S4 P=0.0005  S3 vs S4 P=0.0001 |  |  |  |
| SKCM | S1 vs S2 P=0.27  S1 vs S3 P=0.55  S1 vs S4 P=0.73  S2 vs S3 P=0.14  S2 vs S4 P=0.58  S3 vs S4 P=0.47 |  |  | TP53 mutant vs TP53 NON MUTANT P=0.002 |
| STAD | N vs S1 P=0.14  N vs S2 P=1×10^-6^  N s S3 P=9×10^-10^  N vs S4 P=0.001  S1 vs S2 P=0.7  S1 vs S3 P=0.57  S1 vs S4 P=0.94  S2 vs S3 P=0.55  S2 vs S4 P=0.6  S3 vs S4 P=0.4 | N vs Grade 1 P=0.09  N vs Grade 2 P=1×10^-7^  N vs Grade 3 P= 1×10^-9^  Grade 1 vs Grade 2 P=0.4  Grade 1 vs Grade 3 P=0  Grade 2 vs Grade 3 P=0.9 | N vs Adenocarcinoma (NOS) P=8×10^-9^  N vs Adenocarcinoma (Diffuse) P=1×10^-5^  N vs Intestinal Adenocarcinoma (NOS) P=1×10^-5^  N vs IntestinalAdenocarcinoma (Tubular) P=1×10^-6^  N vs Intestinaladenocarcinoma (Mucinous) P=4 ×10^-5^  N vs IntestinalAdenocarcinoma (Papillary) P=0.033 | N vs TP53 mutant P=1×10^-6^  N vs TP53 Non mutant P=3×10^-11^  TP53 Mutant vs TP53Non mutant P=0.02 |
| TGCT |  |  | Seminoma vs Non seminoma P=0.0008 |  |
| TH CA | N vs S1 P=0.004  N vs S2 P=0.59  N vs S3 P=0.0001  N vs S4 P=1×10^-5^  S1 vs S2 P=0.03  S1 vs S3 P=0.19  S1 vs S4 P=0.01  S2 vs S3 P=0.003  SS2 vs S4 P=0.0004  S3 vs S4 P=0.2 |  | Classical Vs Tall P=0.005  Classical vs Follicular P=0.3  Classical vs Other P=0.1  Classical vs Normal P=0.01  Tall vs Follicular P=0.04  Tall vs other P=0.8  Tall vs Normal P=5×10^-5^  Follicular vs other P=0.26  Follicular vs normal P=0.008  Other vs Normal P=0.014 |  |
| UCEC | N vs S1 P=4×10^-7^  N vs S2 P=1×10^-7^  N vs S3 0.0007  N vs S4 P=0.14  S1 vs S2 P=0.27  S1 vs S3 P=0.06  S1 vs S4 P=0.55  S2 vs S3 P=0.01  S2 vs S4 P=0.34  S3 vs S4 P=0.98 |  | Normal vs Endometrioid P=1×10^-7^  N vs Serous P=0.003  N vs Mixed serous and 3ndometriod P=0.056  Endometrioid vs Serous P=0.07  Endometrioid vs Mixed serous and Endometrioid P=0.16  Serous vs Mixed Serous and Endometrioid P=0.95 | Normal vs TP53 mutant P=1×10^-6^  Normal vs TP53 Non mutant P=1×10^-6^  TP53 Mutant vs TP53 Non mutant P=0.8 |
| OV | S2 vs S3 P=0.01  S2 vs S4 P=0.1  S3 vs S4 P=0.15 |  |  |  |
| PAAD | N vs S1 P=0.23  N vs S2 P=0.04 N vs S3 P=0.002  N vs S4 P=0.09  S1 vs S2 P=0.48  S1 vs S3 P=0.13  S1 vs S4 P=0.64  S2 vs S3 P=0.15  S2 vs S4 P=0.94  S3 vs S4 P=0.25 | N vs Grade 1 P=0.9  N vs Grade 2 P=0.8  N vs Grade 3 P=0.83  N vs Grade 4 P=1  Grade1 vs Grade 2 P=0.04  Grade 1 vs Grade 3 P=0.03  Grade 1 vs Grade 4 P=0.9  Garde 2 vs Grade 3 P=0.6  Grade 2 vs Grade 4 P=0.8  Grade 3 vs Garde 4 P=0.84 |  | N vs TP53 Mutant P=0.02  N vs TP53 Non mutant P=0.4  TP53 mutant vs TP53 Nonmutant P=0.0006 |
| PRAD |  |  |  | N vs TP53 mutant P=1×10^-5^  N vs TP53 Non mutant P=4×10^-6^  TP53 Mutant vs TP53 Non mutant P=0.042 |
| READ | N vs stage 1 P=0.53  N vs Stage 2 P=0.4  N vs Stage 3 P=0.42  N vs Stage 4 P=0.89  Stage 1 vs Stage 2 P=0.04  Stage 1 vs Stage 3 P=0.026  Stage 1 vs Stage 4 P=0.49  Stage 2 vs Stage 3 P=0.86  Stage 2 vs Stage 4 P=0.22  Stage 3 vs Stage 4 P=0.17 |  |  |  |

Table S2: This table highlights protein alterations, mutation types, copy number variations, and allele frequencies in TCGA cancer samples having USP22-related mutation events across several cancer types.

| Sample ID | Cancer Type Detailed | Protein Change | Mutation Type | Copy | Allele Freq (T) | Mut in Sample |
| --- | --- | --- | --- | --- | --- | --- |
| [TCGA-EW-A2FS-01](https://www.cbioportal.org/patient?sampleId=TCGA-EW-A2FS-01&studyId=brca_tcga_pan_can_atlas_2018) | Breast Invasive Ductal Carcinoma | ***EXOC7-USP22 Fusion*** | **Fusion** | **Amp** |  | 27 |
| [TCGA-AR-A1AT-01](https://www.cbioportal.org/patient?sampleId=TCGA-AR-A1AT-01&studyId=brca_tcga_pan_can_atlas_2018) | Breast Invasive Lobular Carcinoma | ***RASL10B-USP22 Fusion*** | **Fusion** |  |  | 27 |
| [TCGA-A8-A097-01](https://www.cbioportal.org/patient?sampleId=TCGA-A8-A097-01&studyId=brca_tcga_pan_can_atlas_2018) | Breast Invasive Ductal Carcinoma | ***USP22-MMS22L Fusion*** | **Fusion** | **Amp** |  | 89 |
| [TCGA-BH-A0DD-01](https://www.cbioportal.org/patient?sampleId=TCGA-BH-A0DD-01&studyId=brca_tcga_pan_can_atlas_2018) | Breast Invasive Ductal Carcinoma | ***USP22-MYO19 Fusion*** | **Fusion** | **Amp** |  | 21 |
| [TCGA-D8-A27N-01](https://www.cbioportal.org/patient?sampleId=TCGA-D8-A27N-01&studyId=brca_tcga_pan_can_atlas_2018) | Breast Invasive Ductal Carcinoma | ***USP22-SLC39A11 Fusion*** | **Fusion** | **Gain** |  | 41 |
| [TCGA-A8-A06Z-01](https://www.cbioportal.org/patient?sampleId=TCGA-A8-A06Z-01&studyId=brca_tcga_pan_can_atlas_2018) | Breast Invasive Ductal Carcinoma | ***USP22-SPACA3 Fusion*** | **Fusion** | **ShallowDel** |  | 70 |
| [TCGA-PC-A5DL-01](https://www.cbioportal.org/patient?sampleId=TCGA-PC-A5DL-01&studyId=sarc_tcga_pan_can_atlas_2018) | Leiomyosarcoma | ***USP22-EPN2 Fusion*** | **Fusion** | **Amp** |  |  |
| [TCGA-CH-5790-01](https://www.cbioportal.org/patient?sampleId=TCGA-CH-5790-01&studyId=prad_tcga_pan_can_atlas_2018) | Prostate Adenocarcinoma | ***TMEM170A-USP22 Fusion*** | **Fusion** | **ShallowDel** |  | 20 |
| [TCGA-HC-A6AN-01](https://www.cbioportal.org/patient?sampleId=TCGA-HC-A6AN-01&studyId=prad_tcga_pan_can_atlas_2018) | Prostate Adenocarcinoma | ***USP22-ATP2A3 Fusion*** | **Fusion** | Diploid |  | 30 |
| [TCGA-V1-A8ML-01](https://www.cbioportal.org/patient?sampleId=TCGA-V1-A8ML-01&studyId=prad_tcga_pan_can_atlas_2018) | Prostate Adenocarcinoma | ***USP22-RNF167 Fusion*** | **Fusion** | **ShallowDel** |  | 10 |
| [TCGA-46-3769-01](https://www.cbioportal.org/patient?sampleId=TCGA-46-3769-01&studyId=lusc_tcga_pan_can_atlas_2018) | Lung Squamous Cell Carcinoma | ***USP22-CCDC144NL Fusion*** | **Fusion** | Diploid |  | 811 |
| [TCGA-13-0884-01](https://www.cbioportal.org/patient?sampleId=TCGA-13-0884-01&studyId=ov_tcga_pan_can_atlas_2018) | Serous Ovarian Cancer | ***L205Cfs*51*** | **FS del** | **ShallowDel** | 0.37 | 112 |
| [TCGA-41-2572-01](https://www.cbioportal.org/patient?sampleId=TCGA-41-2572-01&studyId=gbm_tcga_pan_can_atlas_2018) | Glioblastoma Multiforme | ***C61Y*** | **Missense** | Diploid | 0.41 | 48 |
| [TCGA-16-0848-01](https://www.cbioportal.org/patient?sampleId=TCGA-16-0848-01&studyId=gbm_tcga_pan_can_atlas_2018) | Glioblastoma Multiforme | ***T363M*** | **Missense** | Diploid | 0.16 | 332 |
| [TCGA-33-4533-01](https://www.cbioportal.org/patient?sampleId=TCGA-33-4533-01&studyId=lusc_tcga_pan_can_atlas_2018) | Lung Squamous Cell Carcinoma | ***L465Vfs*5*** | **FS del** | Diploid | 0.58 | 268 |
| [TCGA-56-8201-01](https://www.cbioportal.org/patient?sampleId=TCGA-56-8201-01&studyId=lusc_tcga_pan_can_atlas_2018) | Lung Squamous Cell Carcinoma | ***S146L*** | **Missense** | **Gain** | 0.1 | 149 |
| [TCGA-56-8626-01](https://www.cbioportal.org/patient?sampleId=TCGA-56-8626-01&studyId=lusc_tcga_pan_can_atlas_2018) | Lung Squamous Cell Carcinoma | ***T361M*** | **Missense** | **ShallowDel** | 0.18 | 226 |
| [TCGA-85-8352-01](https://www.cbioportal.org/patient?sampleId=TCGA-85-8352-01&studyId=lusc_tcga_pan_can_atlas_2018) | Lung Squamous Cell Carcinoma | ***G284E*** | **Missense** | **Gain** | 0.32 | 251 |
| [TCGA-E7-A85H-01](https://www.cbioportal.org/patient?sampleId=TCGA-E7-A85H-01&studyId=blca_tcga_pan_can_atlas_2018) | Bladder Urothelial Carcinoma | ***R420W*** | **Missense** | **Gain** | 0.24 | 383 |
| [TCGA-G3-AAV5-01](https://www.cbioportal.org/patient?sampleId=TCGA-G3-AAV5-01&studyId=lihc_tcga_pan_can_atlas_2018) | Hepatocellular Carcinoma | ***S217N*** | **Missense** | **ShallowDel** | 0.41 | 76 |
| [TCGA-DD-AAD1-01](https://www.cbioportal.org/patient?sampleId=TCGA-DD-AAD1-01&studyId=lihc_tcga_pan_can_atlas_2018) | Hepatocellular Carcinoma | ***G109S*** | **Missense** | **ShallowDel** | 0.07 | 112 |
| [TCGA-2Y-A9GX-01](https://www.cbioportal.org/patient?sampleId=TCGA-2Y-A9GX-01&studyId=lihc_tcga_pan_can_atlas_2018) | Hepatocellular Carcinoma | ***S215N*** | **Missense** | Diploid | 0.09 | 55 |
| [TCGA-DD-AACD-01](https://www.cbioportal.org/patient?sampleId=TCGA-DD-AACD-01&studyId=lihc_tcga_pan_can_atlas_2018) | Hepatocellular Carcinoma | ***Y425D*** | **Missense** | Diploid | 0.13 | 86 |
| [TCGA-HC-A76X-01](https://www.cbioportal.org/patient?sampleId=TCGA-HC-A76X-01&studyId=prad_tcga_pan_can_atlas_2018) | Prostate Adenocarcinoma | ***K159**** | **Nonsense** | Diploid | 0.38 | 29 |
| [TCGA-XK-AAIW-01](https://www.cbioportal.org/patient?sampleId=TCGA-XK-AAIW-01&studyId=prad_tcga_pan_can_atlas_2018) | Prostate Adenocarcinoma | ***R133**** | **Nonsense** | Diploid | 0.36 | 6230 |
| [TCGA-V1-A9OA-01](https://www.cbioportal.org/patient?sampleId=TCGA-V1-A9OA-01&studyId=prad_tcga_pan_can_atlas_2018) | Prostate Adenocarcinoma | ***F428S*** | **Missense** | Diploid | 0.14 | 17 |
| [TCGA-BP-5198-01](https://www.cbioportal.org/patient?sampleId=TCGA-BP-5198-01&studyId=kirc_tcga_pan_can_atlas_2018) | Renal Clear Cell Carcinoma | ***C383Y*** | **Missense** | **ShallowDel** | 0.24 | 69 |
| [TCGA-AJ-A3EL-01](https://www.cbioportal.org/patient?sampleId=TCGA-AJ-A3EL-01&studyId=ucec_tcga_pan_can_atlas_2018) | Uterine Endometrioid Carcinoma | ***E92K*** | **Missense** | Diploid | 0.41 | 7385 |
| [TCGA-AP-A056-01](https://www.cbioportal.org/patient?sampleId=TCGA-AP-A056-01&studyId=ucec_tcga_pan_can_atlas_2018) | Uterine Endometrioid Carcinoma | ***R133Q*** | **Missense** | Diploid | 0.23 | 8231 |
| [TCGA-EY-A1GD-01](https://www.cbioportal.org/patient?sampleId=TCGA-EY-A1GD-01&studyId=ucec_tcga_pan_can_atlas_2018) | Uterine Endometrioid Carcinoma | ***R133Q*** | **Missense** | Diploid | 0.47 | 1076 |
| [TCGA-AX-A063-01](https://www.cbioportal.org/patient?sampleId=TCGA-AX-A063-01&studyId=ucec_tcga_pan_can_atlas_2018) | Uterine Endometrioid Carcinoma | ***T363M*** | **Missense** | Diploid | 0.42 | 1001 |
| [TCGA-EY-A215-01](https://www.cbioportal.org/patient?sampleId=TCGA-EY-A215-01&studyId=ucec_tcga_pan_can_atlas_2018) | Uterine Endometrioid Carcinoma | ***Y258C*** | **Missense** | Diploid | 0.12 | 4646 |
| [TCGA-AX-A05W-01](https://www.cbioportal.org/patient?sampleId=TCGA-AX-A05W-01&studyId=ucec_tcga_pan_can_atlas_2018) | Uterine Endometrioid Carcinoma | ***R133**** | **Nonsense** | Diploid | 0.06 | 66 |
| [TCGA-AX-A2HG-01](https://www.cbioportal.org/patient?sampleId=TCGA-AX-A2HG-01&studyId=ucec_tcga_pan_can_atlas_2018) | Uterine Endometrioid Carcinoma | ***R201Q*** | **Missense** | Diploid | 0.43 | 861 |
| [TCGA-A5-A0G2-01](https://www.cbioportal.org/patient?sampleId=TCGA-A5-A0G2-01&studyId=ucec_tcga_pan_can_atlas_2018) | Uterine Serous Carcinoma/Uterine Papillary Serous Carcinoma | ***K129N*** | **Missense** | Diploid | 0.23 | 25698 |
| [TCGA-AP-A0LM-01](https://www.cbioportal.org/patient?sampleId=TCGA-AP-A0LM-01&studyId=ucec_tcga_pan_can_atlas_2018) | Uterine Endometrioid Carcinoma | ***V404I*** | **Missense** | Diploid | 0.3 | 13203 |
| [TCGA-EO-A22R-01](https://www.cbioportal.org/patient?sampleId=TCGA-EO-A22R-01&studyId=ucec_tcga_pan_can_atlas_2018) | Uterine Endometrioid Carcinoma | ***V404I*** | **Missense** | Diploid | 0.35 | 12771 |
| [TCGA-D1-A17Q-01](https://www.cbioportal.org/patient?sampleId=TCGA-D1-A17Q-01&studyId=ucec_tcga_pan_can_atlas_2018) | Uterine Endometrioid Carcinoma | ***D496N*** | **Missense** | Diploid | 0.5 | 5948 |
| [TCGA-EO-A22X-01](https://www.cbioportal.org/patient?sampleId=TCGA-EO-A22X-01&studyId=ucec_tcga_pan_can_atlas_2018) | Uterine Endometrioid Carcinoma | ***D496N*** | **Missense** | Diploid | 0.4 | 9421 |
| [TCGA-AP-A059-01](https://www.cbioportal.org/patient?sampleId=TCGA-AP-A059-01&studyId=ucec_tcga_pan_can_atlas_2018) | Uterine Endometrioid Carcinoma | ***R176H*** | **Missense** | Diploid | 0.25 | 10944 |
| [TCGA-DI-A1BU-01](https://www.cbioportal.org/patient?sampleId=TCGA-DI-A1BU-01&studyId=ucec_tcga_pan_can_atlas_2018) | Uterine Mixed Endometrial Carcinoma | ***R176H*** | **Missense** | Diploid | 0.32 | 7632 |
| [TCGA-AX-A05W-01](https://www.cbioportal.org/patient?sampleId=TCGA-AX-A05W-01&studyId=ucec_tcga_pan_can_atlas_2018) | Uterine Endometrioid Carcinoma | ***E525Sfs*74*** | **FS del** | Diploid | 0.28 | 66 |
| [TCGA-D1-A103-01](https://www.cbioportal.org/patient?sampleId=TCGA-D1-A103-01&studyId=ucec_tcga_pan_can_atlas_2018) | Uterine Endometrioid Carcinoma | ***E391K*** | **Missense** | Diploid | 0.39 | 6851 |
| [TCGA-D1-A103-01](https://www.cbioportal.org/patient?sampleId=TCGA-D1-A103-01&studyId=ucec_tcga_pan_can_atlas_2018) | Uterine Endometrioid Carcinoma | ***L200M*** | **Missense** | Diploid | 0.38 | 6851 |
| [TCGA-D1-A17B-01](https://www.cbioportal.org/patient?sampleId=TCGA-D1-A17B-01&studyId=ucec_tcga_pan_can_atlas_2018) | Uterine Endometrioid Carcinoma | ***R367P*** | **Missense** | Diploid | 0.1 | 286 |
| [TCGA-A5-A0G2-01](https://www.cbioportal.org/patient?sampleId=TCGA-A5-A0G2-01&studyId=ucec_tcga_pan_can_atlas_2018) | Uterine Serous Carcinoma/Uterine Papillary Serous Carcinoma | ***F516L*** | **Missense** | Diploid | 0.16 | 25698 |
| [TCGA-A5-A0G2-01](https://www.cbioportal.org/patient?sampleId=TCGA-A5-A0G2-01&studyId=ucec_tcga_pan_can_atlas_2018) | Uterine Serous Carcinoma/Uterine Papillary Serous Carcinoma | ***X315_splice*** | **Splice** | Diploid | 0.08 | 25698 |
| [TCGA-AJ-A23O-01](https://www.cbioportal.org/patient?sampleId=TCGA-AJ-A23O-01&studyId=ucec_tcga_pan_can_atlas_2018) | Uterine Endometrioid Carcinoma | ***Y524**** | **Nonsense** | Diploid | 0.33 | 860 |
| [TCGA-AP-A051-01](https://www.cbioportal.org/patient?sampleId=TCGA-AP-A051-01&studyId=ucec_tcga_pan_can_atlas_2018) | Uterine Endometrioid Carcinoma | ***V191M*** | **Missense** | Diploid | 0.1 | 8312 |
| [TCGA-B5-A3FA-01](https://www.cbioportal.org/patient?sampleId=TCGA-B5-A3FA-01&studyId=ucec_tcga_pan_can_atlas_2018) | Uterine Endometrioid Carcinoma | ***V191M*** | **Missense** | Diploid | 0.33 | 9645 |
| [TCGA-AP-A051-01](https://www.cbioportal.org/patient?sampleId=TCGA-AP-A051-01&studyId=ucec_tcga_pan_can_atlas_2018) | Uterine Endometrioid Carcinoma | ***I179T*** | **Missense** | Diploid | 0.09 | 8312 |
| [TCGA-AP-A1DK-01](https://www.cbioportal.org/patient?sampleId=TCGA-AP-A1DK-01&studyId=ucec_tcga_pan_can_atlas_2018) | Uterine Endometrioid Carcinoma | ***X368_splice*** | **Splice** | Diploid | 0.3 | 10303 |
| [TCGA-AP-A1DK-01](https://www.cbioportal.org/patient?sampleId=TCGA-AP-A1DK-01&studyId=ucec_tcga_pan_can_atlas_2018) | Uterine Endometrioid Carcinoma | ***L341P*** | **Missense** | Diploid | 0.14 | 10303 |
| [TCGA-AX-A1CE-01](https://www.cbioportal.org/patient?sampleId=TCGA-AX-A1CE-01&studyId=ucec_tcga_pan_can_atlas_2018) | Uterine Endometrioid Carcinoma | ***K87N*** | **Missense** | Diploid | 0.46 | 11442 |
| [TCGA-AX-A2HC-01](https://www.cbioportal.org/patient?sampleId=TCGA-AX-A2HC-01&studyId=ucec_tcga_pan_can_atlas_2018) | Uterine Endometrioid Carcinoma | ***R367Q*** | **Missense** | Diploid | 0.35 | 12979 |
| [TCGA-B5-A1MX-01](https://www.cbioportal.org/patient?sampleId=TCGA-B5-A1MX-01&studyId=ucec_tcga_pan_can_atlas_2018) | Uterine Endometrioid Carcinoma | ***N348D*** | **Missense** | Diploid | 0.12 | 5687 |
| [TCGA-B5-A1MX-01](https://www.cbioportal.org/patient?sampleId=TCGA-B5-A1MX-01&studyId=ucec_tcga_pan_can_atlas_2018) | Uterine Endometrioid Carcinoma | ***V273M*** | **Missense** | Diploid | 0.34 | 5687 |
| [TCGA-B5-A3FA-01](https://www.cbioportal.org/patient?sampleId=TCGA-B5-A3FA-01&studyId=ucec_tcga_pan_can_atlas_2018) | Uterine Endometrioid Carcinoma | ***R368Q*** | **Missense** | Diploid | 0.6 | 9645 |
| [TCGA-D1-A1NS-01](https://www.cbioportal.org/patient?sampleId=TCGA-D1-A1NS-01&studyId=ucec_tcga_pan_can_atlas_2018) | Uterine Endometrioid Carcinoma | ***A270T*** | **Missense** | Diploid | 0.31 | 290 |
| [TCGA-EO-A22U-01](https://www.cbioportal.org/patient?sampleId=TCGA-EO-A22U-01&studyId=ucec_tcga_pan_can_atlas_2018) | Uterine Endometrioid Carcinoma | ***A263T*** | **Missense** | Diploid | 0.77 | 13822 |
| [TCGA-EO-A22U-01](https://www.cbioportal.org/patient?sampleId=TCGA-EO-A22U-01&studyId=ucec_tcga_pan_can_atlas_2018) | Uterine Endometrioid Carcinoma | ***R153W*** | **Missense** | Diploid | 0.27 | 13822 |
| [TCGA-EY-A215-01](https://www.cbioportal.org/patient?sampleId=TCGA-EY-A215-01&studyId=ucec_tcga_pan_can_atlas_2018) | Uterine Endometrioid Carcinoma | ***R164G*** | **Missense** | Diploid | 0.2 | 4646 |
| [TCGA-FI-A2D5-01](https://www.cbioportal.org/patient?sampleId=TCGA-FI-A2D5-01&studyId=ucec_tcga_pan_can_atlas_2018) | Uterine Endometrioid Carcinoma | ***G83C*** | **Missense** | Diploid | 0.11 | 13853 |
| [TCGA-73-4659-01](https://www.cbioportal.org/patient?sampleId=TCGA-73-4659-01&studyId=luad_tcga_pan_can_atlas_2018) | Lung Adenocarcinoma | ***K144R*** | **Missense** | **ShallowDel** | 0.27 | 192 |
| [TCGA-05-4396-01](https://www.cbioportal.org/patient?sampleId=TCGA-05-4396-01&studyId=luad_tcga_pan_can_atlas_2018) | Lung Adenocarcinoma | ***G182W*** | **Missense** | **ShallowDel** | 0.1 | 1287 |
| [TCGA-L5-A8NM-01](https://www.cbioportal.org/patient?sampleId=TCGA-L5-A8NM-01&studyId=esca_tcga_pan_can_atlas_2018) | Esophageal Adenocarcinoma | ***G347D*** | **Missense** | Diploid | 0.24 | 1429 |
| [TCGA-EE-A20H-06](https://www.cbioportal.org/patient?sampleId=TCGA-EE-A20H-06&studyId=skcm_tcga_pan_can_atlas_2018) | Cutaneous Melanoma | ***R176L*** | **Missense** | Diploid | 0.51 | 518 |
| [TCGA-D3-A51R-06](https://www.cbioportal.org/patient?sampleId=TCGA-D3-A51R-06&studyId=skcm_tcga_pan_can_atlas_2018) | Cutaneous Melanoma | ***P337L*** | **Missense** | Diploid | 0.45 | 592 |
| [TCGA-D9-A148-06](https://www.cbioportal.org/patient?sampleId=TCGA-D9-A148-06&studyId=skcm_tcga_pan_can_atlas_2018) | Cutaneous Melanoma | ***K129N*** | **Missense** | **ShallowDel** | 0.1 | 839 |
| [TCGA-BF-A1Q0-01](https://www.cbioportal.org/patient?sampleId=TCGA-BF-A1Q0-01&studyId=skcm_tcga_pan_can_atlas_2018) | Cutaneous Melanoma | ***L465M*** | **Missense** |  | 0.1 | 2628 |
| [TCGA-EE-A29Q-06](https://www.cbioportal.org/patient?sampleId=TCGA-EE-A29Q-06&studyId=skcm_tcga_pan_can_atlas_2018) | Cutaneous Melanoma | ***Q490K*** | **Missense** | Diploid | 0.08 | 615 |
| [TCGA-EE-A2ME-06](https://www.cbioportal.org/patient?sampleId=TCGA-EE-A2ME-06&studyId=skcm_tcga_pan_can_atlas_2018) | Cutaneous Melanoma | ***G234**** | **Nonsense** | Diploid | 0.12 | 522 |
| [TCGA-W3-AA1V-06](https://www.cbioportal.org/patient?sampleId=TCGA-W3-AA1V-06&studyId=skcm_tcga_pan_can_atlas_2018) | Cutaneous Melanoma | ***P162L*** | **Missense** | Diploid | 0.26 | 4559 |
| [TCGA-BR-7851-01](https://www.cbioportal.org/patient?sampleId=TCGA-BR-7851-01&studyId=stad_tcga_pan_can_atlas_2018) | Intestinal Type Stomach Adenocarcinoma | ***R176C*** | **Missense** | Diploid | 0.19 | 1308 |
| [TCGA-BR-4280-01](https://www.cbioportal.org/patient?sampleId=TCGA-BR-4280-01&studyId=stad_tcga_pan_can_atlas_2018) | Stomach Adenocarcinoma | ***S482R*** | **Missense** | Diploid | 0.36 | 705 |
| [TCGA-BR-4361-01](https://www.cbioportal.org/patient?sampleId=TCGA-BR-4361-01&studyId=stad_tcga_pan_can_atlas_2018) | Stomach Adenocarcinoma | ***M213I*** | **Missense** | Diploid | 0.08 | 2290 |
| [TCGA-VQ-A8P2-01](https://www.cbioportal.org/patient?sampleId=TCGA-VQ-A8P2-01&studyId=stad_tcga_pan_can_atlas_2018) | Mucinous Stomach Adenocarcinoma | ***R98Q*** | **Missense** | Diploid | 0.34 | 6364 |
| [TCGA-B7-5816-01](https://www.cbioportal.org/patient?sampleId=TCGA-B7-5816-01&studyId=stad_tcga_pan_can_atlas_2018) | Diffuse Type Stomach Adenocarcinoma | ***G174D*** | **Missense** | Diploid | 0.1 | 1200 |
| [TCGA-MX-A5UJ-01](https://www.cbioportal.org/patient?sampleId=TCGA-MX-A5UJ-01&studyId=stad_tcga_pan_can_atlas_2018) | Tubular Stomach Adenocarcinoma | ***T393I*** | **Missense** | Diploid | 0.11 | 1295 |
| [TCGA-BH-A0HF-01](https://www.cbioportal.org/patient?sampleId=TCGA-BH-A0HF-01&studyId=brca_tcga_pan_can_atlas_2018) | Breast Invasive Ductal Carcinoma | ***R210H*** | **Missense** |  | 0.16 | 832 |
| [TCGA-BH-A0HF-01](https://www.cbioportal.org/patient?sampleId=TCGA-BH-A0HF-01&studyId=brca_tcga_pan_can_atlas_2018) | Breast Invasive Ductal Carcinoma | ***R208K*** | **Missense** |  | 0.16 | 832 |
| [TCGA-D8-A1XK-01](https://www.cbioportal.org/patient?sampleId=TCGA-D8-A1XK-01&studyId=brca_tcga_pan_can_atlas_2018) | Breast Invasive Ductal Carcinoma | ***L245P*** | **Missense** | **ShallowDel** | 0.44 | 971 |
| [TCGA-A8-A081-01](https://www.cbioportal.org/patient?sampleId=TCGA-A8-A081-01&studyId=brca_tcga_pan_can_atlas_2018) | Breast Invasive Ductal Carcinoma | ***D460N*** | **Missense** | **ShallowDel** | 0.09 | 108 |
| [TCGA-DM-A1HA-01](https://www.cbioportal.org/patient?sampleId=TCGA-DM-A1HA-01&studyId=coadread_tcga_pan_can_atlas_2018) | Colon Adenocarcinoma | ***R419Q*** | **Missense** | Diploid | 0.53 | 133 |
| [TCGA-AA-3949-01](https://www.cbioportal.org/patient?sampleId=TCGA-AA-3949-01&studyId=coadread_tcga_pan_can_atlas_2018) | Mucinous Adenocarcinoma of the Colon and Rectum | ***A269T*** | **Missense** | Diploid | 0.23 | 1164 |
| [TCGA-AA-A01R-01](https://www.cbioportal.org/patient?sampleId=TCGA-AA-A01R-01&studyId=coadread_tcga_pan_can_atlas_2018) | Mucinous Adenocarcinoma of the Colon and Rectum | ***V68I*** | **Missense** | Diploid | 0.32 | 1621 |
| [TCGA-CA-6717-01](https://www.cbioportal.org/patient?sampleId=TCGA-CA-6717-01&studyId=coadread_tcga_pan_can_atlas_2018) | Mucinous Adenocarcinoma of the Colon and Rectum | ***E143D*** | **Missense** | Diploid | 0.18 | 9586 |
| [TCGA-CA-6717-01](https://www.cbioportal.org/patient?sampleId=TCGA-CA-6717-01&studyId=coadread_tcga_pan_can_atlas_2018) | Mucinous Adenocarcinoma of the Colon and Rectum | ***R133Q*** | **Missense** | Diploid | 0.14 | 9586 |
| [TCGA-AD-5900-01](https://www.cbioportal.org/patient?sampleId=TCGA-AD-5900-01&studyId=coadread_tcga_pan_can_atlas_2018) | Mucinous Adenocarcinoma of the Colon and Rectum | ***R210C*** | **Missense** | Diploid | 0.26 | 1474 |
| [TCGA-BA-A6DJ-01](https://www.cbioportal.org/patient?sampleId=TCGA-BA-A6DJ-01&studyId=hnsc_tcga_pan_can_atlas_2018) | Head and Neck Squamous Cell Carcinoma | ***D202Y*** | **Missense** | Diploid | 0.26 | 232 |
| [TCGA-CN-6997-01](https://www.cbioportal.org/patient?sampleId=TCGA-CN-6997-01&studyId=hnsc_tcga_pan_can_atlas_2018) | Head and Neck Squamous Cell Carcinoma | ***I381M*** | **Missense** | Diploid | 0.5 | 197 |
| [TCGA-D6-6516-01](https://www.cbioportal.org/patient?sampleId=TCGA-D6-6516-01&studyId=hnsc_tcga_pan_can_atlas_2018) | Head and Neck Squamous Cell Carcinoma | ***V404A*** | **Missense** | Diploid | 0.27 | 1524 |
| [TCGA-CV-5434-01](https://www.cbioportal.org/patient?sampleId=TCGA-CV-5434-01&studyId=hnsc_tcga_pan_can_atlas_2018) | Head and Neck Squamous Cell Carcinoma | ***A128T*** | **Missense** | **Gain** | 0.38 | 131 |
| [TCGA-VS-A958-01](https://www.cbioportal.org/patient?sampleId=TCGA-VS-A958-01&studyId=cesc_tcga_pan_can_atlas_2018) | Cervical Squamous Cell Carcinoma | ***S318C*** | **Missense** | **ShallowDel** | 0.39 | 746 |

**Table S3:** Hazard ratio of the overall survival rate

| ***hazard ratio ( ±95% confidence interval)*** | **Altered group** | **Unaltered group** |
| --- | --- | --- |
| Altered group | 1.000 (0.734-1.362) | 1.285 (1.030-1.602) |
| Unaltered group | 0.778 (0.624-0.971) | 1.000 (0.954-1.048) |

**Table S4**: Survival plot summary

|  | **Number of Cases, Total** | **Number of Events** | **Median Months Overall (95% CI)** |
| --- | --- | --- | --- |
| Altered group | **228** | **63** | **107.18 (90.38 - NA)** |
| Unaltered group | **10575** | **3450** | **78.41 (72.23 - 83.24)** |

**Table S5**: Hazards ratio for diseases specific survival

| ***hazard ratio ( ±95% confidence interval)*** | **Altered group** | **Unaltered group** |
| --- | --- | --- |
| Altered group | **1.000 (0.692-1.446)** | **1.388 (1.066-1.807)** |
| Unaltered group | **0.721 (0.553-0.938)** | **1.000 (0.945-1.058)** |

**Table S6**: Summary of diseases specific plot

|  | **Number of Cases, Total** | **Number of Events** | **Median Months Disease-specific (95% CI)** |
| --- | --- | --- | --- |
| Altered group | 222 | 41 | 168.26 (103.26 - NA) |
| Unaltered group | 10036 | 2393 | 133.74 (117.40 - 152.81) |
